# Mutant *INS*-Gene Induced Diabetes of Youth: Proinsulin Cysteine Residues Impose Dominant-Negative Inhibition on Wild-Type Proinsulin Transport

**DOI:** 10.1371/journal.pone.0013333

**Published:** 2010-10-11

**Authors:** Ming Liu, Leena Haataja, Jordan Wright, Nalinda P. Wickramasinghe, Qing-Xin Hua, Nelson F. Phillips, Fabrizio Barbetti, Michael A. Weiss, Peter Arvan

**Affiliations:** 1 Division of Metabolism, Endocrinology and Diabetes, University of Michigan Medical Center, Ann Arbor, Michigan, United States of America; 2 Department of Biochemistry, Case Western Reserve University, Cleveland, Ohio, United States of America; 3 Laboratory of Molecular Endocrinology and Metabolism, Bambino Gesù Children's Hospital, Scientific Institute (IRCCS), Rome, Italy; 4 Department of Internal Medicine, University of Tor Vergata, Rome, Italy; La Jolla Institute of Allergy and Immunology, United States of America

## Abstract

Recently, a syndrome of ***M***utant ***I*** *NS*-gene-induced ***D***iabetes of ***Y***outh ***(MIDY***, derived from one of 26 distinct mutations) has been identified as a cause of insulin-deficient diabetes, resulting from expression of a misfolded mutant proinsulin protein in the endoplasmic reticulum (ER) of insulin-producing pancreatic beta cells. Genetic deletion of one, two, or even three alleles encoding insulin in mice does not necessarily lead to diabetes. Yet *MIDY* patients are *INS*-gene heterozygotes; inheritance of even one *MIDY* allele, causes diabetes. Although a favored explanation for the onset of diabetes is that insurmountable ER stress and ER stress response from the mutant proinsulin causes a net loss of beta cells, in this report we present three surprising and interlinked discoveries. First, in the presence of *MIDY* mutants, an increased fraction of wild-type proinsulin becomes recruited into nonnative disulfide-linked protein complexes. Second, regardless of whether *MIDY* mutations result in the loss, or creation, of an extra unpaired cysteine within proinsulin, Cys residues in the mutant protein are nevertheless essential in causing intracellular entrapment of co-expressed wild-type proinsulin, blocking insulin production. Third, while each of the *MIDY* mutants induces ER stress and ER stress response; ER stress and ER stress response alone appear insufficient to account for blockade of wild-type proinsulin. While there is general agreement that ultimately, as diabetes progresses, a significant loss of beta cell mass occurs, the early events described herein precede cell death and loss of beta cell mass. We conclude that the molecular pathogenesis of *MIDY* is initiated by perturbation of the disulfide-coupled folding pathway of wild-type proinsulin.

## Introduction

Insulinopathies classically have been described as rare monogenic causes of adult diabetes mellitus caused by point mutations leading to selective amino-acid substitution within the mutant insulin molecule or its precursor, involving impaired sorting to secretory granules, endoproteolytic conversion to insulin, or binding to insulin receptors [1], [2], [3]. More recent are numerous reports of insulin-deficient diabetes caused by heterozygous mutations in the insulin gene [4], [5], [6], [7], [8]. These mutations account for a significant subset of cases of permanent neonatal-onset diabetes mellitus [9], a syndrome referred to as ***M***utant ***I*** *NS*-gene induced ***D***iabetes of ***Y***outh (*MIDY*) [10], [11].

The discovery of *MIDY* has stimulated renewed interest in the earliest steps of the insulin biosynthesis pathway [12]. Upon delivery to the endoplasmic reticulum (ER), preproinsulin undergoes co-translational translocation with cleavage of the signal peptide. Folding is initiated upon translocation into the ER lumen; acquisition of proinsulin tertiary structure [10] is coupled to the catalyzed oxidation of the hormone's three evolutionarily conserved disulfide bonds [13]. Proinsulin can form zinc-independent dimers and undergo transport to the Golgi complex where zinc-stabilized hexamers are thought to form before their proteolytic processing in newly-forming insulin secretory granules [14].

*MIDY* mutations impair proinsulin folding; such products of the mutant *INS*-gene allele are deficient in producing insulin [4], [5], [15], [16], [17]. Genetic deficiency of insulin expression can cause diabetes [18], but *MIDY* patients are heterozygotes and *INS*-gene haploinsufficiency is not itself a sufficient basis for diabetes, at least not in mice [19], [20], [21], [22]. Thus, it is not clear why *MIDY* patients — all of whom co-express wild-type proinsulin alongside the mutant proinsulin— should develop diabetes. A prevailing thought is that chronic ER stress with unremitting ER stress response activation triggers pancreatic beta cell death with a loss of pancreatic beta cell mass [23], [24], [25]. Indeed, there is little dispute that ultimately, after diabetes progresses in both humans and animal models, there is a loss of beta cell mass [26]; yet debate continues about whether impaired beta cell function precedes or follows the loss of beta cell mass [27]. *Akita* mice transmit heterozygous inheritance of a single *MIDY* mutant proinsulin-C(A7)Y allele that causes autosomal dominant diabetes [28]. Though ultimately, *Akita* diabetic mice end up with few if any beta cells [29], a recent study reports that at the time of initial onset of the hyperglycemia of *MIDY*, these animals have expansion of their beta cell mass from beta cell hyperplasia within each islet [30].

In the present study we examine the initial molecular mechanism of *MIDY* and find that it involves misfolded mutant copies of nascent proinsulin recruiting wild-type proinsulin into misfolded disulfide-linked protein complexes and thereby inhibiting wild-type insulin production.

## Results

### MIDY mutants, blocked in export, engage in disulfide-linked protein complexes

Following cleavage of the preproinsulin signal peptide (residues 1–24), three domains of proinsulin follow sequentially (Table S1): the B-chain (residues 1–30), flanking dibasic cleavage sites plus C-peptide (-2; residues 1–31; +2), and A-chain (residues 1–21). Proper folding of proinsulin requires formation of three evolutionarily-conserved disulfide bridges (Table S1, *upper left diagram*): C(B7)-C(A7), C(B19)-C(A20), and C(A6)-C(A11), while the C-peptide ordinarily has no cysteines and remains disordered [31], [32]. Three insulinopathies, F(B24)S, F(B25)L, and V(A3)L, are classically associated with adult-onset diabetes (Table S1). Although proinsulin-F(B24)S was less efficient in export, all three were secreted significantly (Figure S1A, B). Further, the human proinsulin-G(C28)R variant, reported as a possible cause of disease [7], was exported normally (Figure S1A, B), and led to normal insulin production in the Min6 pancreatic beta cell line (not shown) whereas *MIDY* mutants [11] (a subset from the list in Table S1) were largely blocked in secretion (Fig. S1A, B).

**Figure 1:**
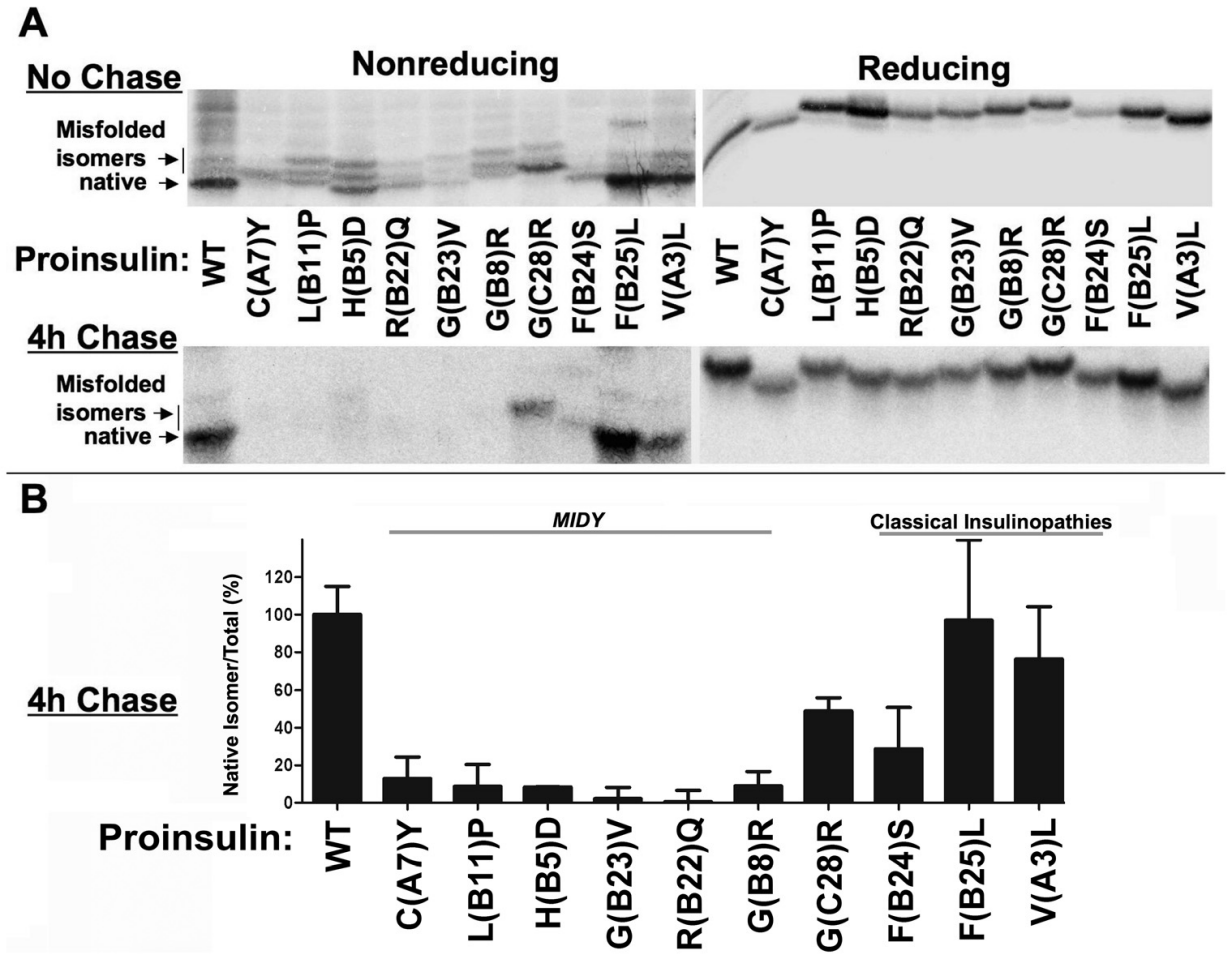
*MIDY* proinsulins form abnormally increased amounts of disulfide-linked protein complexes. 293T cells were transfected with vector expressing preproinsulin wild type (‘WT’) or preproinsulin missense mutants in which the described mutation is within the B-chain, C-peptide, or A-chain. At 40 h post-transfection, cells were pulse-labeled with ^35^S-amino acids for 1 h and then chased for the times indicated. For completeness, chase media and cell lysates were mixed, but none of the *MIDY* proinsulins are appreciably secreted (see Figure S1). **A.** At both time points, samples were immunoprecipitated with anti-insulin followed by Tris-tricine-urea-SDS-PAGE under both nonreducing (gels on left) and reducing conditions (gels on right), followed by fluorography. **B**. The fractional recovery of the native isoform of newly-synthesized proinsulin (fastest migrating band under nonreduced conditions) at 4 h of chase was compared against the recovery from the same sample under reducing conditions (considered to represent total at that chase time). The relative recovery for proinsulin-WT served as a positive control (ie, set to 100%). Results are expressed as mean ± s.d. from two independent experiments.

Many *MIDY* mutants involve gain or loss of a cysteinyl residue [4], [5], [6], [7], [8], and proinsulin-C(A7)Y (causing diabetes in the *Akita* mouse) is the most studied of these [15], [23], [33], [34], [35], [36]. Although Izumi et al. reported no evidence for abnormally increased intracellular levels of abnormally disulfide-bonded protein in cells expressing proinsulin-C(A7)Y [34], we re-visited this question with a simple assay to quantify recovery by Tris-tricine-urea-SDS-PAGE of the native proinsulin disulfide isomer band visualized under nonreducing conditions, normalized to total proinsulin visualized under reducing conditions (which abrogates both inter- and intramolecular disulfides and thereby generates a single proinsulin band). While more than half of newly-synthesized wild-type proinsulin [or mutants classically associated with adult-onset diabetes, or the proinsulin-G(C28)R variant] were recovered as the native disulfide isomer, only one third of *MIDY* mutants (35.1%±7.2%) could be recovered as *any* kind of monomeric isomer (Fig. 1A *upper panels*). By 4 h after synthesis, some loss of misfolded proinsulin may have occurred by ER-associated degradation; more importantly, of residual *MIDY* proinsulin, the relative recovery became vanishingly small under nonreduced (compared to reduced) conditions (Fig. 1A lower panel, quantified in Fig. 1B). These data demonstrate that *MIDY* mutants engage in abnormally increased intermolecular disulfide-linked protein complexes as well as abnormal intramolecular disulfide isomers. As described further (below), this aberrant behavior also occurs *in vivo* as it is found in pancreatic islets of *Akita* mice, a mouse model of *MIDY*.

### Designing a mutant proinsulin that cannot engage in intermolecular disulfide-linked protein complexes

**Figure 2:**
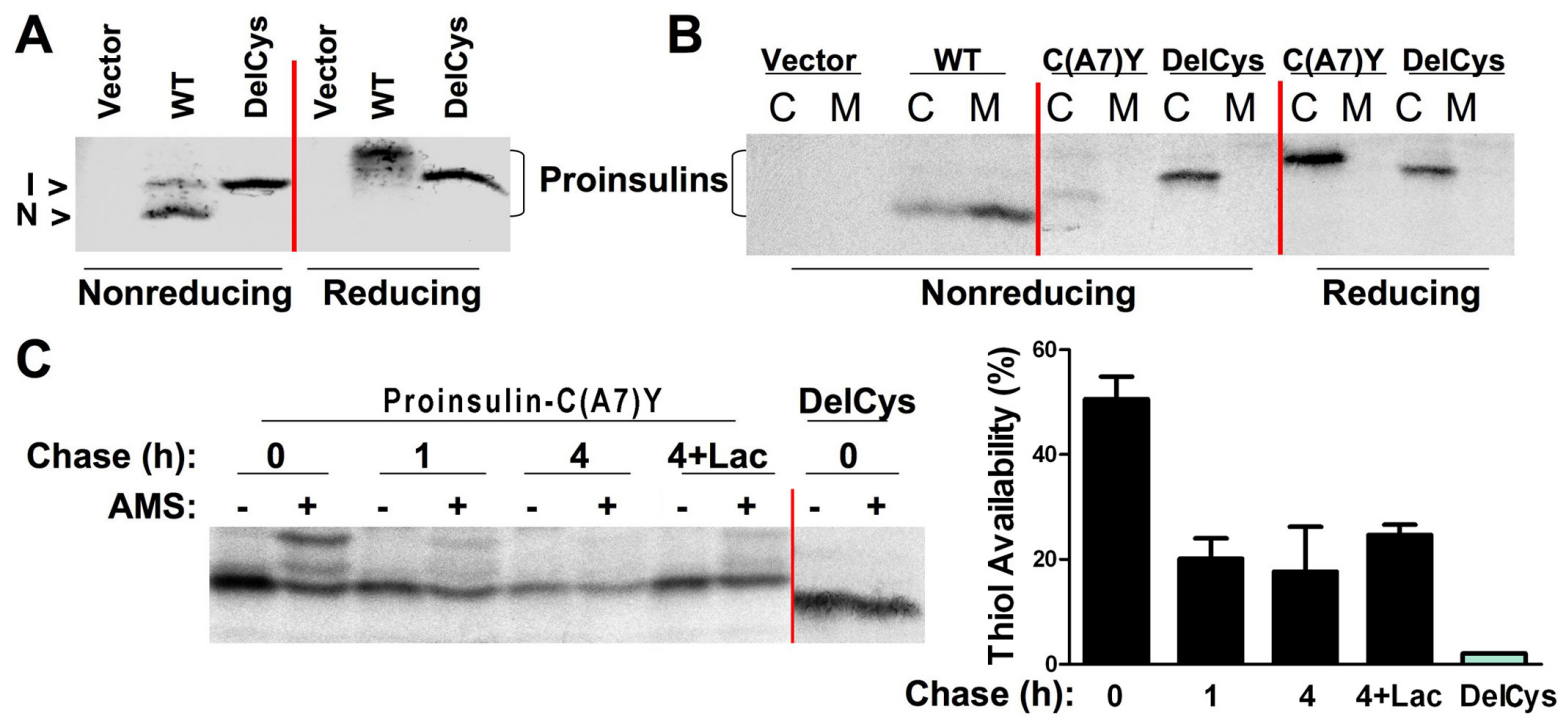
Misfolded proinsulin with or without free cysteine thiols. **A.** 293T cells transiently expressing mouse proinsulin-WT (‘WT’) or proinsulin-DelCys (‘DelCys’) were pulse-labeled with ^35^S-amino acids for 1 h without chase. Cells were lysed and immunoprecipitated with anti-insulin, followed by Tris-tricine-urea-SDS-PAGE under both nonreducing and reducing conditions as indicated, followed by fluorography. “N” = proinsulin with native disulfide pairs; “I” = proinsulin disulfide isomer(s). Note that proinsulin-DelCys has identical gel mobility under nonreducing and reducing conditions. **B.** 293T cells transiently expressing empty vector, proinsulin-WT, proinsulin-C(A7)Y, or proinsulin-DelCys were pulse-labeled with ^35^S-amino acids for 1 h and chased for 1 h. Cell lysates (‘C’) and chase media (‘M’) were immunoprecipitated with anti-insulin and analyzed as in **A**. **C.** 293T cells transiently expressing proinsulin-C(A7)Y or proinsulin-DelCys were pulse-labeled with ^35^S-amino acids for 1 h and chased in complete medium for the times indicated. For one sample, 10 µM lactacystin (‘Lac’) was added to the chase medium bathing transfected cells. At each chase time, cells were lysed, immunoprecipitated with anti-insulin, and the immunoprecipitates incubated with or without AMS as described in Methods, and analyzed by reducing Tris-Tricine-urea-SDS-PAGE and fluorography. As a fraction of all proinsulin bands recovered per lane, thiol-consumed (nonreactive) and thiol-available (AMS reactive) subfractions of newly-synthesized proinsulin-C(A7)Y were quantified by scanning densitometry; the average and range from two independent such experiments is shown at right. Note that proinsulin-DelCys has no reactivity with AMS.

To design a misfolded, nonsecreted mutant proinsulin incapable of engaging in intermolecular disulfide bonds, we introduced missense substitutions for all 6 Cys residues to create proinsulin-DelCys [or simply “DelCys” bearing the *Akita*-like C(A7)Y but also containing C(B7)S, C(B19)S, C(A20)S, C(A6)M, and C(A11)M mutations that permit metabolic labeling with ^35^S-methionine]. Not surprisingly, DelCys was completely defective for protein secretion from cells to medium, but unlike proinsulin-C(A7)Y, DelCys was quantitatively recovered upon nonreducing tris-tricine-urea-SDS-PAGE, and its gel mobility was unchanged under nonreducing *versus* reducing conditions (Figs. 2A, B).

Bearing no Cys residues, DelCys should not be able to be alkylated with AMS, a maleimide derivative that irreversibly adds 0.5 kD of molecular mass for each thiol modified [37]. We compared the (lack of) alkylation of DelCys to that of the proinsulin-C(A7)Y *MIDY* mutant with its odd number of Cys residues. At different times after synthesis, immunoprecipitates of metabolically labeled proinsulin mutants were divided in half and either reacted or mock-treated with AMS. Newly-synthesized proinsulin-C(A7)Y should have at least one free thiol available unless it uses an unpaired cysteine to engage in an intermolecular disulfide bond, in which case it cannot react with AMS. Immediately after synthesis, approximately half of the C(A7)Y molecules had already consumed available cysteine thiols (Fig. 2C *gel at left; quantified at right*). Over the next 4 h, the remaining proinsulin-C(A7)Y had consumed nearly all free thiols, Fig. 2C). Addition of the proteasome inhibitor lactacystin during these 4 h increased final recovery, but the proinsulin-C(A7)Y still engaged in disulfide-linked complexes as judged by a loss of free thiols (Fig. 2C). By contrast, DelCys showed no reactivity with AMS at any time (Fig. 2C and data not shown), consistent with an inability to engage in intermolecular disulfide-linked protein complexes. Further, we confirmed AMS modification of proinsulin-C(A7)Y in metabolically-labeled islets from *Akita* mice, whereas no AMS modification of labeled proinsulin was observed in islets from wild-type mice (not shown).

### Dominant-negative inhibition of wild-type proinsulin trafficking is a distinguishing feature of MIDY mutants

**Figure 3:**
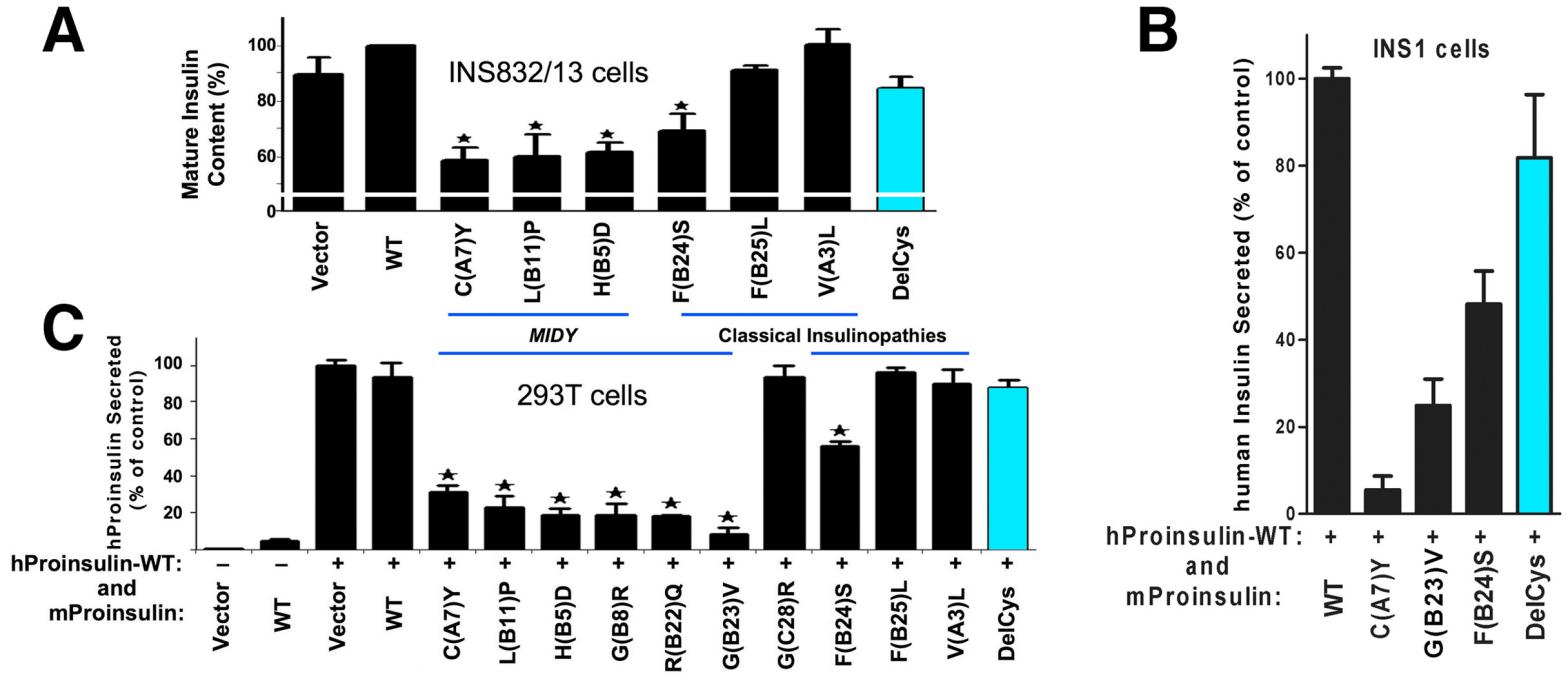
Effect of mutant proinsulins on insulin storage derived from co-expressed nonmutant proinsulin in pancreatic beta cells (and effect of mutant proinsulins on proinsulin export in 293T cells). **A.** INS832/13 cells, which co-store human insulin in secretory granules, were transiently transfected with pCMS-GFP to co-express mouse preproinsulins (as indicated) and cytosolic GFP. Transfected cells were recovered after 48 h by fluorescence-activated cell sorting and were extracted with acid-ethanol. A human insulin-specific radioimmunoassay was used to measure hormone storage in mature secretory granules, normalized to cell number. When mouse proinsulin-WT was expressed, the amount of human insulin stored served as a positive control (ie, set to 100%). Results shown are the mean ± s.d. in at least three independent experiments. *p<0.05 compared with mouse proinsulin-WT. **B.** INS-1 cells were co-transfected with wild-type human preproinsulin and wild-type or mutant mouse preproinsulin. Transfected cells were incubated for 28 h with fresh medium before collection for measurement of secreted human insulin using human insulin specific radioimmunoassay. **C.** 293T cells were co-transfected to express 1) human proinsulin-WT and co-express 2) mouse proinsulin-WT or missense mutants in which the described mutation is within the B-chain, C-peptide, A-chain, or proinsulin-DelCys at a plasmid ratio of 1 **:** 2. Beginning at 24 h post-transfection, cells were incubated for 16 h with high-glucose DMEM plus 10% FBS. Media were collected and a human proinsulin-specific radioimmunoassay was used to measure secretion of co-expressed human proinsulin-WT. When mouse proinsulin was replaced by empty vector, the amount of human proinsulin-WT secretion served as a positive control (ie, set to 100%). Medium collected from 293T cells expressing only mouse proinsulin-WT served as a negative control for the specificity of the human proinsulin radioimmunoassay (while independent measurements not shown proved ample secretion of rodent proinsulin-WT in these samples). Results are expressed as mean ± s.d. from at least three independent experiments. *p<0.05 compared with mouse proinsulin-WT.

We previously reported that proinsulin-C(A7)Y can engage co-expressed nonmutant proinsulin in protein complexes within the ER [36]; such a mechanism could underlie the dominant-negative inhibition of wild-type proinsulin transport that has been observed by us and others [15], [16], [29]. We wished to compare the extent to which wild-type proinsulin trafficking from the ER could be blocked by *MIDY* mutants that have substitutions in residues other than a cysteine. In the rat INS832/13 beta cell line that co-synthesizes wild-type human insulin, we analyzed by human-specific radioimmunoassay the content of mature human insulin (produced in secretory granules [38]) as an indicator of the ability of human proinsulin to reach post-ER compartments. Like for proinsulin-C(A7)Y, expression *in trans* of mouse proinsulin-H(B5)D or L(B11)P inhibited production of mature human insulin despite the fact that these *MIDY* mutant proinsulins neither added nor lost a cysteine residue. Importantly, despite being itself misfolded and entrapped in the secretory pathway (Fig. 2B), DelCys was largely ineffective in blocking production of wild-type human insulin from a co-expressed allele (Fig. 3A, *right*), and unlike *MIDY* mutants [such as proinsulin-C(A7)Y and G(B23)V], DelCys could not effectively inhibit secretion of co-expressed wild-type human insulin (Fig. 3B). [As expected, proinsulin mutants F(B25)L and V(A3)L, classically associated with adult-onset diabetes, were unable to block human insulin production, whereas proinsulin-F(B24)S exhibited an intermediate phenotype (Fig. 3A)].

To ensure that the observed results did not reflect mutant proinsulin action within the secretory granule compartment [16], [17], we used a human-specific proinsulin radioimmunoassay in 293T cells that make neither insulin nor secretory granules, to examine secretion of wild-type human proinsulin co-expressed in the presence of mouse wild-type or mutant proinsulins. Although none bears an extra or missing Cys residue, each of the *MIDY* proinsulins tested [bearing substitutions H(B5)D, G(B8)R, L(B11)P, R(B22)Q, or G(B23)V] severely inhibited secretion of co-expressed human proinsulin-WT. Despite being even more severely unstructured, proinsulin-DelCys showed no significant dominant-negative behavior (Fig. 3C), which corresponds with its inability to form aberrant intermolecular disulfide bonds (Fig. 2). Together, the data strongly suggest that dominant-negative effects on the export of co-expressed proinsulin-WT does not require an odd number of Cys residues in the primary structure of *MIDY* mutants [e.g., H(B5)D, G(B8)R, L(B11)P, R(B22)Q, G(B23)V] yet cysteine exposure in the proinsulin ***folding pathway*** is linked to dominant-negative disease.

**Figure 4 pone-0013333-g004:**
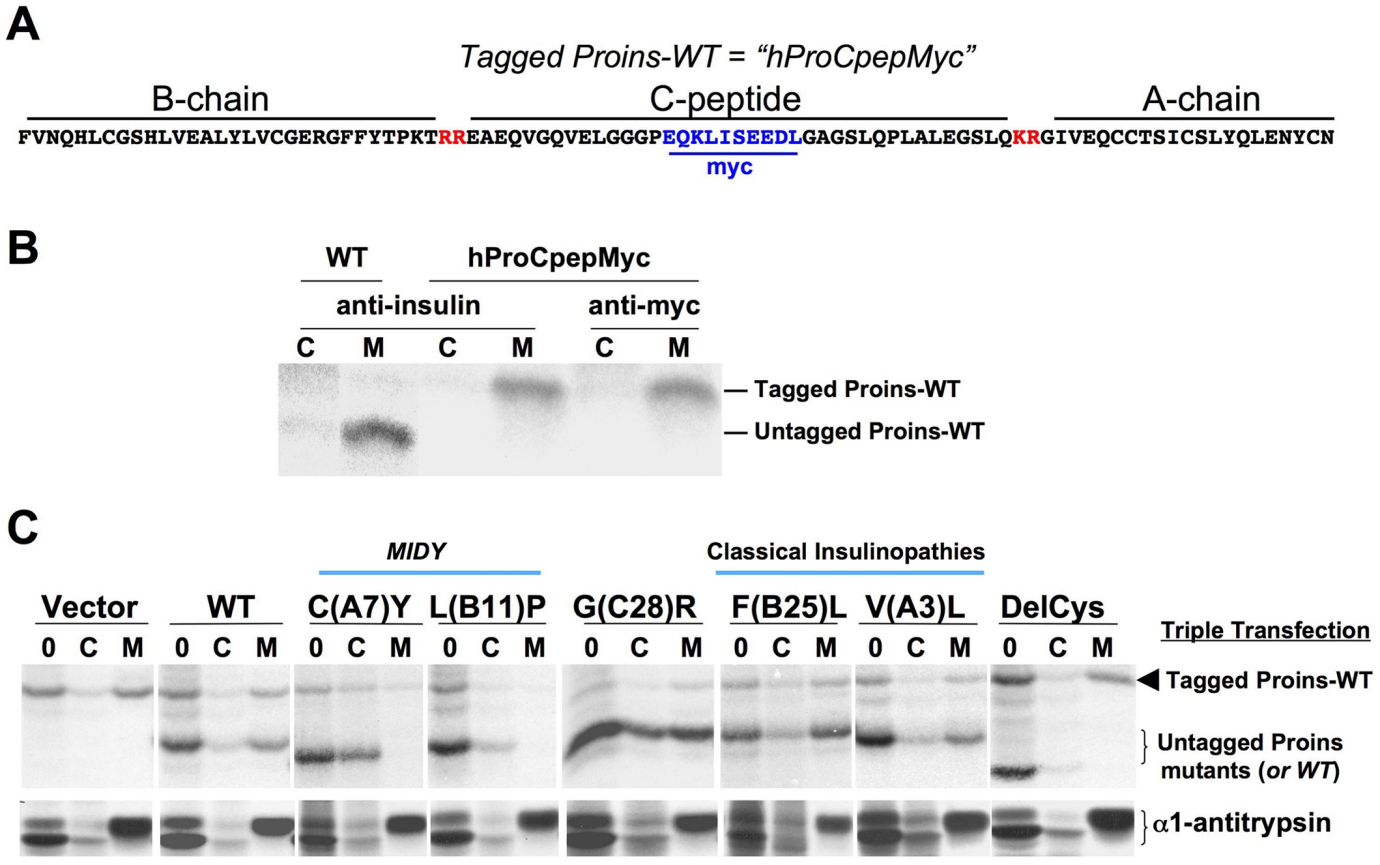
Co-expression of mutant proinsulins with proinsulin-WT and an unrelated secretory protein demonstrates protein-specific dominant-negative inhibition of proinsulin export. **A**. A Myc-epitope tag was inserted into the C-peptide of human proinsulin-WT to form the construct known as hProCpepMyc, whose peptide sequence is shown. **B**. 293T cells were transfected to express either human proinsulin or hProCpepMyc. At 48 h post-transfection, cells were pulse-labeled with ^35^S-amino acids for 1 h and chased for 1 h. Cell lysates (‘C’) and chase media (‘M’) were immunoprecipitated with anti-insulin or anti-myc antibodies. There was no recovery of untagged proinsulin-WT with anti-myc antibodies in either cells or medium (not shown). Immunoprecipitates were analyzed by nonreducing Tris-tricine-urea-SDS-PAGE and fluorography. Note that hProCpepMyc is secreted efficiently and is recovered equally with anti-insulin and anti-myc immunoprecipitation, but the protein has a higher apparent molecular mass than proinsulin-WT. **C**. 293T cells were triply co-transfected to express hProCpepMyc, mouse proinsulins (as indicated) and α_1_-antitrypsin at a plasmid ratio of 2 **:** 4 **:** 1, respectively. At 48 h post-transfection, cells were pulse-labeled with ^35^S-amino acids for 1 h and either lysed at the zero chase time (‘0’) or chased for 3 h. Cell lysates (‘C’) and chase media (‘M’) were immunoprecipitated with anti-insulin (upper set of gels) and anti-α_1_-antitrypsin (lower set of gels). Proinsulin immunoprecipitates were analyzed by Tris-tricine-urea-SDS-PAGE while α_1_-antitrypsin was analyzed by conventional SDS-PAGE, both under reducing conditions. Note that while the secretion efficiency of untagged mutant proinsulin and tagged proinsulin-WT varied, a_1_-antitrypsin was efficiently secreted in every case.

In order to simultaneously follow wild-type proinsulin bystander molecules while monitoring general ER export, we co-expressed proinsulins of distinct molecular mass (that could be resolved by SDS-PAGE) while simultaneously examining α_1_-antitrypsin [an unrelated secretory protein that is well expressed in pancreatic beta-cells [39]]. To introduce a small difference in molecular mass into wild-type human proinsulin, a myc-epitope tag was bioengineered into the C-peptide to create ‘hProCpepMyc’ (also called ‘tagged Proins-WT’, Fig. 4A). Tagged proinsulin-WT is efficiently secreted from cells and is immunoprecipitated equally well with anti-insulin or anti-myc antibodies (Fig. S1C, and Fig. 4B). 293T cells expressing tagged proinsulin-WT, untagged proinsulin mutant, and α_1_-antitrypsin were split into two wells for pulse-labeling. One well was lysed at the zero chase time (lanes marked “0”) and the second chased before cell lysates (“C”) and media (“M”) were immunoprecipitated with anti-insulin and anti-α_1_-antitrypsin, respectively (Fig. 4C). Notably, when plasmid encoding untagged proinsulin was eliminated and replaced by empty vector, only one proinsulin band was immunoprecipitated, corresponding to tagged proinsulin-WT, and the newly-synthesized proinsulin was predominantly secreted (Fig. 4C *upper left*). When tagged proinsulin-WT was co-expressed either with untagged proinsulin-WT or the G(C28)R variant or mutants classically associated with adult-onset diabetes, both sets of proinsulins were predominantly secreted (Fig. 4C). However, *MIDY* mutants with or without an odd number of cysteines [C(A7)Y or L(B11)P] were not only defective for secretion but also inhibited secretion of tagged proinsulin-WT (Fig. 4C). Importantly, co-expressed α_1_-antitrypsin continued to acquire Golgi sugar modifications and to exhibit efficient secretion from these same cells expressing *MIDY* mutants (Fig. 4C *bottom panels*). These data indicate that early toxic effects of *MIDY* proinsulin expression act via blockade of Proins-WT transport prior to loss of cells from cell death, because the cells are actively synthesizing proteins and because the general secretory pathway is functionally operational despite blockade of proinsulin-WT transport. Moreover, untagged DelCys, while completely blocked in its secretion, could not block the predominant secretion of tagged proinsulin-WT, underscoring yet again that at least one Cys residue is needed for the dominant-negative phenotype (Fig. 4C).

**Figure 5:**
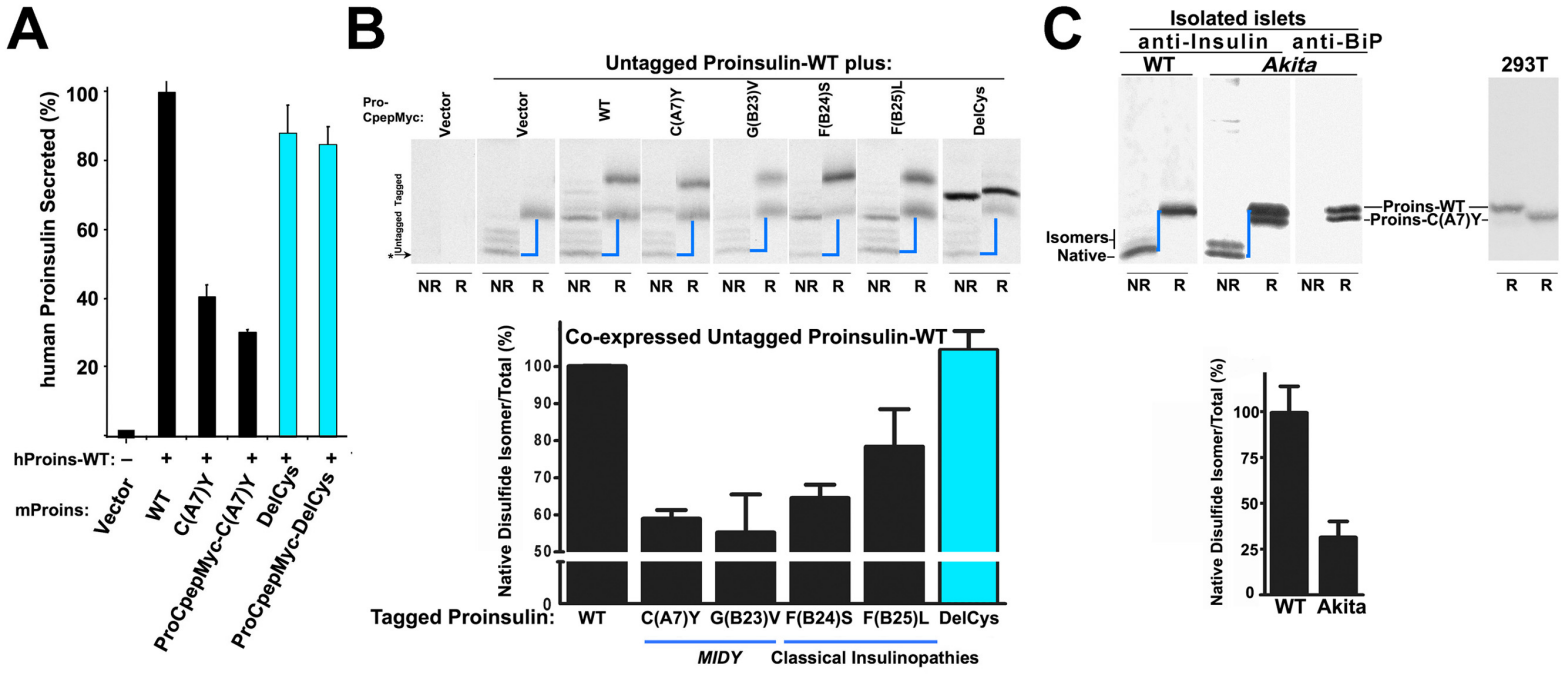
*MIDY* proinsulins cause co-expressed proinsulin-WT to become abnormally engaged in disulfide-linked protein complexes. **A**. 293T cells were co-transfected to express human proinsulin-WT and co-express untagged or Myc-tagged proinsulin-C(A7)Y or proinsulin-DelCys, using a plasmid ratio of 1 **:** 2. At 24 h post-transfection, the cells were incubated in fresh high glucose DMEM containing 10% FBS for 16 h and media were analyzed using a human proinsulin-specific radioimmunoassay. Medium collected from cells transfected with empty vectors served as a negative control; medium collected from cells transfected human proinsulin-WT and mouse proinsulin-WT served as a positive control (ie, set to 100%). Results are expressed as mean ± s.d. from at least three independent measurements. Note that presence of the Myc-tag neither caused nor prevented dominant-negative inhibition of co-expressed human proinsulin-WT. **B.** 293T cells were co-transfected to express untagged human proinsulin-WT and co-express Myc-tagged proinsulin-WT or missense mutants, using a plasmid ratio of 1 **:** 1. At 48 h post-transfection, cells were pulse-labeled with ^35^S-amino acids for 1 h, lysed, and immunoprecipitated with anti-insulin followed by analysis under nonreducing (‘NR’) and reducing (‘R’) conditions as indicated (note that NR and R lanes were run at opposite ends of the gel and upon fluorography were spliced after imaging to juxtapose the two conditions for each sample). The fractional recovery of co-expressed untagged human proinsulin-WT under nonreducing conditions was compared against the recovery of the same untagged proinsulin-WT in these samples under reducing conditions (in the Figure, the bands in question connected by a blue line). With the relative recovery of untagged human proinsulin-WT co-expressed in the presence of tagged proinsulin-WT serving as a positive control (ie, set to 100%), the bar graph below quantifies the bands (and the dominant-negative effect) from three replicate experiments. **C.** Islets isolated from wild-type control mice were pulse-labeled with ^35^S-amino acids for 20 min without chase. The lysates were immunoprecipitated with anti-insulin, followed by analysis under nonreducing (‘NR’) and reducing (‘R’) conditions as indicated. Islets isolated from male *Akita* mice were similarly labeled, lysed, and the lysates divided for immunoprecipitation with anti-insulin or co-precipitation with anti-BiP. Under reducing conditions, the wild-type and mutant gene products of *Akita* islets could be separated and their full recovery quantified. Under nonreducing conditions, the recovery of the native disulfide isomer of wild-type proinsulin was calculated relative to total recovery of the wild-type gene product under reducing conditions. Note that the wild-type translation product is the fastest band on the nonreducing gel and the slowest band on the reducing gel (also note that NR and R lanes were run at opposite ends of the gel and upon fluorography were spliced after imaging to juxtapose the two conditions for each sample). The bar graph below quantifies this ratio for wild-type proinsulin in *Akita* islets relative to that obtained in wild-type (‘WT’) islets ± s.d., from three independent experiments (p<0.05 compared to wild-type islets). At right, recombinant human proinsulin-WT or -C(A7)Y were expressed and labeled in 293T cells to serve as molecular mass markers for the positions of these proteins under reducing conditions.

To determine if the myc-epitope tag contributes to impaired trafficking of co-expressed wild-type proinsulin, we compared results when transferring the tag to the mutant partner. Regardless of tag, the proinsulin-C(A7)Y mutant inhibited wild-type proinsulin secretion; and regardless of tag, DelCys could not inhibit wild-type proinsulin secretion (Fig. 5A). It is thus irrelevant which partner has the myc tag.

If dominant-negative inhibition of proinsulin export by co-expressed *MIDY* mutants is caused by recruitment of proinsulin-WT into abnormal disulfide-linked protein complexes, then a selective defect in recovery of native proinsulin-WT would be expected under nonreducing conditions. Indeed, we found that recovery of the native isomer of untagged wild-type proinsulin (detected under nonreducing–‘NR’ conditions) was inhibited the most in cells co-expressing *MIDY* proinsulins (compare bands connected by blue lines in Fig. 5B *; data from three independent experiments are quantified in the graph below the gel*). To examine the pathophysiological significance of this finding, we examined the islets of male *Akita* mice [in which it is well established that at early stages of the disease, islet beta-cells remain abundant and proinsulin translation per islet is either normal or actually increased despite that insulin production per islet is dramatically decreased [29], [30], [33], [36], [40]. Using a modification of the Tris-tricine-urea-SDS-PAGE gel system [41], we could resolve the products of the wild-type and mutant proinsulin alleles under reducing conditions. With this method (upper panels of Fig. 5C), we determined from three independent experiments that the single proinsulin-C(A7)Y allele (driven by the *Ins2* promoter) in *Akita* islets accounts for 36.1%±4.8% of newly-synthesized translation product immunoprecipitable with anti-insulin (i.e., 63.9% wild-type translation product). In normal control islets (i.e., 100% wild-type translation product), absolute recovery of the native disulfide isomer of proinsulin under nonreduced conditions was incomplete (79.5% of total), consistent with the observation that unsuccessful protein production occurs significantly even in the absence of any misfolding-inducing mutation [42]. However, even when normalized to this control level of misfolding (Fig. 5C bottom panel), in *Akita* islets recovery of the native disulfide isomer from the three wild-type alleles was still less than half of that expected. Thus, the inefficient delivery of the wild-type proinsulin gene product for packaging into secretory granules [36] can be explained by recruitment of wild-type proinsulin into misfolded disulfide-linked protein complexes (Fig. 5C).

In islet beta-cells, BiP associates with proinsulin [43] preferentially to misfolded forms [44]. When newly-synthesized proinsulin from pulse-labeled *Akita* islets was co-precipitated with BiP, almost nothing was recovered under nonreducing conditions (Fig. 5C). However, under reducing conditions, not only mutant proinsulin but also wild-type proinsulin was strongly recovered (Fig. 5C upper panel). Thus, both from cultured cells and from isolated islets of *Akita* mice, the data in Figs. 3–5 directly demonstrate that in *MIDY*, the presence of the mutant gene product increases misfolding of the wild-type gene product, which can account for the onset of insulin deficiency triggering diabetes [28].

### Structural features of a MIDY mutant

Proinsulin exhibits an insulin-like core but a disordered C-peptide domain that renders *in vitro* structural analyses difficult. Therefore, structures and stabilities of mutant insulins are typically used to provide insight into the role of individual residues within the corresponding proinsulins. We wished to examine how *MIDY* mutations might affect the efficiency of peptide chain assembly *in vitro* (the ability of isolated B- and A-chains to undergo specific disulfide pairing), a method that reflects the ability to properly align cysteine pairings between B- and A-chains. A mutation classically associated with adult-onset diabetes, i.e., V(A3)L, does not impair insulin chain assembly, and mutations at B24 give rise to only relatively small (2- to 4-fold) decrements in insulin yield [45]. However, upon co-incubation of wild-type A- chain with B-chain bearing the G(B23)V substitution, the insulin product yield (followed by mass spectrometry, reverse-phase high-performance liquid chromatography, or insulin receptor binding) was profoundly inhibited, below the limits of sensititvity (<2% of that of wild-type B-chain), indicating a strong block to interchain disulfide pairing.

**Figure 6:**
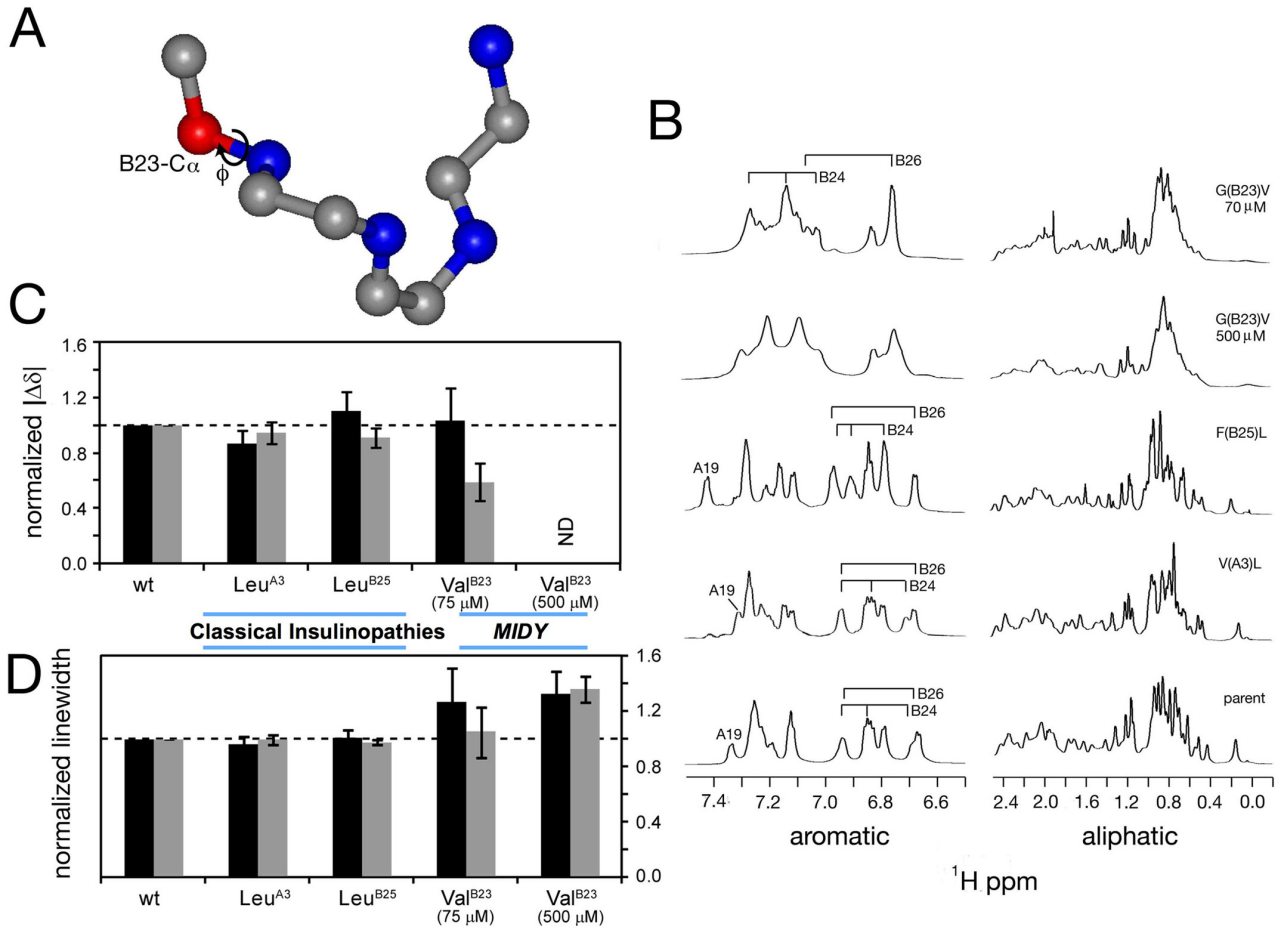
^1^H-NMR analysis of mutant insulins. **A.** Expanded ball-and-stick model of the β-turn from residues B20-B23, highlighting positive φ angle at B23 (circular arrow at left). The C_α_ atom of Gly^B23^ is shown in red; amide nitrogen atoms are shown in blue. **B.** ^1^H-NMR spectra of DKP-insulin analogs in D_2_O (pD 7.0): *top to bottom*, the *MIDY* mutant Val^B23^ (protein concentration 70 µM), Val^B23^ (protein concentration 500 µM); the remaining spectra were obtained at a protein concentration of 500-600 µM: Leu^B25^-DKP-insulin, Leu^A3^-DKP-insulin, and DKP-insulin (parent spectrum). The aliphatic region is shown at right, and aromatic region at left; aromatic spin systems of Phe^B24^ and Tyr^B26^ are indicated. (**C.** and **D.**) Histograms of NMR parameters, highlighting *MIDY*-associated perturbation. For each of DKP-insulin (“wt”), Leu^A3^-DKP-insulin, Leu^B25^-DKP-insulin, and Val^B23^-DKP-insulin, black and gray bars correspond to A-chain probes and B-chain probes, respectively. A-chain probes [A2(γ',δ), A10(γ',δ), and A19(δ,ε)] reflect its helix-turn-helix conformation (see Table S1) whereas B-chain probes [B15(δ), B24(δ,ε,ξ), and B26(δ,ε)] monitor conformation of the C-terminal β-strand and its packing against the central B-chain α-helix (Leu^B15^). **C.** Normalized secondary chemical shifts changes (scs; defined as differences between observed chemical shifts and those tabulated from random-coil values). Significant attenuation of chemical shifts is observed only for the *MIDY* mutant Val^B23^-DKP-insulin. A- and B-chain (black and gray bars, respectively) represent mean scs values of A- and B-chain probe resonances (ND, not determined). **D.** Line widths of the above A-chain or B-chain resonances in corresponding 2D-TOCSY spectra (Figure S2) were normalized by the line widths of corresponding resonances in the wild-type spectrum. Significant peak broadening was observed only in the spectrum of Val^B23^-DKP-insulin, which became more severe as the protein concentration was increased to 500 µM. At 500 µM Val^B23^-DKP-insulin exhibits generalized broadening indicative of protein aggregation; only resonances from A19(δ,ε) and B26(δ,ε) could be used to quantify line widths because the other probe resonances were either too broad or unresolved. B23 bars thus under-estimate extent of resonance broadening.

To proceed further with biophysical studies of insulin-G(B23)V, we exploited a well-studied monomeric, bioactive insulin prefolded with native disulfide pairs already in place (known as ‘DKP-insulin’ [46]) to serve as a polypeptide scaffold for replacement of B23-B30 [47] with a new octapeptide bearing the G(B23)V *MIDY* mutation (Fig. 6A, a ball and stick cartoon highlights local positioning and rotation of residue B23 within the B20-B23 stretch). At high protein concentration, NMR peak broadening for the G(B23)V mutant suggested an increased predisposition for protein aggregation as compared to mutants classically associated with adult-onset diabetes (Fig. 6B). However at low protein concentration, attenuation of chemical shifts observed in 2D-NMR spectra suggested only very limited perturbation in local structure at the carboxyl-terminus of the B-chain (Fig. 6C) and a native-like pattern of local A-chain structure (Fig. 6D). Moreover, when prepared within this prefolded insulin template, the G(B23)V substitution had normal thermodynamic stability based on resistance to chemical denaturation (not shown). The fact that minor perturbation of final insulin structure caused by G(B23)V substitution is greatly outweighed by major defects in the ability to assemble the insulin chains with proper cysteine pairings indicates that this MIDY mutant is blocked primarily in the protein ***folding pathway*** rather than in the stability of the insulin native state.

We also looked for a potential structural basis for the surprising intermediate secretory phenotype and partial dominant-negative phenotype (Figs. 1, 3, 5, and Figure S1) exhibited by the proinsulin-F(B24)S mutant, which heretofore has been associated only with adult-onset diabetes. Notably, we found that whereas the solution structures of insulin-F(B25)L and -V(A3)L are essentially identical to wild-type and cause only trivial decrements in thermodynamic stability (ΔΔG_u_ 0.2 and 0.5 kcal/mole, respectively), computer modeling from NMR spectroscopic data [48] revealed that the F(B24)S mutation results in surprising variation (ie, instability) in the structural coordinates for residues B20-B30 consistent with perturbation C(B19)-C(A20) alignment (Figure S3); and the insulin-F(B24)S mutant exhibits increased sensitivity to guanidinium hydrochloride denaturation (ΔΔG_u_ 1.2 kcal/mole). These results suggest a range of severity of *MIDY* phenotypes that are linked to a continuum of severity of protein folding defects.

### ER stress response caused by MIDY mutants

**Figure 7:**
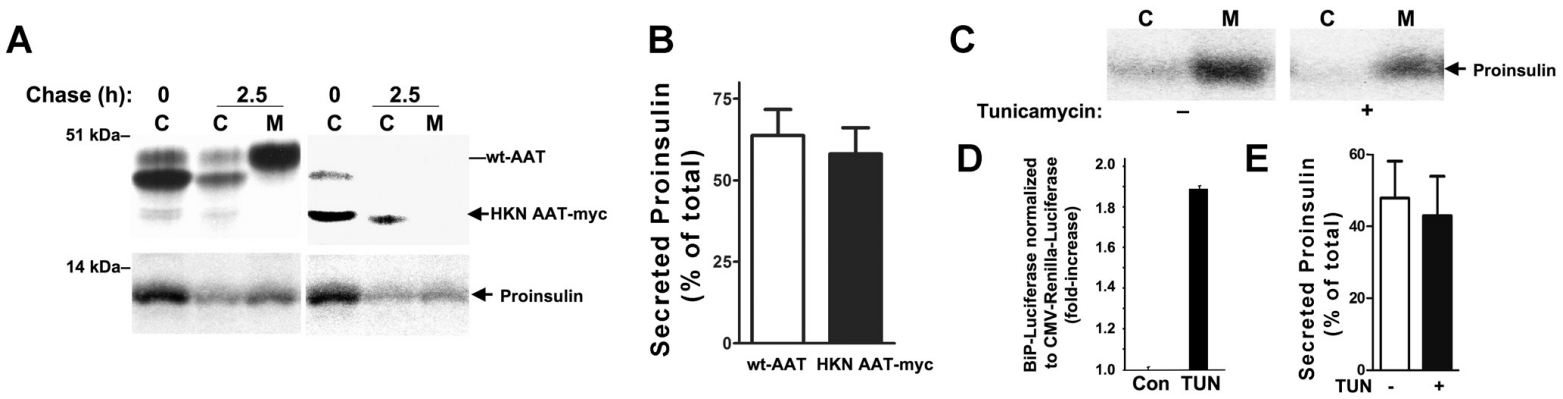
ER stress and ER stress reponse alone do not block wild-type proinsulin secretion. **A.** Wild-type proinsulin secretion from cells co-expressing wild-type α_1_-antitrypsin (“wt-AAT”) or mutant Hong Kong-null α_1_-antitrypsin (“HKN AAT-myc”). 293T cells co-transfected with proinsulin and wt-AAT (left panels) or HKN AAT-myc (right panels) were pulse-labeled with ^35^S-amino acids and chased for the times indicated. Cell lysates and media were immunoprecipitated with anti-AAT (upper panels) or anti-insulin (lower panels) and analyzed by SDS-PAGE and fluorography. During the chase, wt-AAT shifted upwards to a Golgi-glycosylated form and was secreted from cells (“C”) to medium (“M”); proinsulin was secreted in parallel. **B.** Quantification at 2.5 h of chase of the percent of proinsulin recovered in the medium, from three independent experiments. **C.** 293T cells co-transfected with proinsulin, BiP-luciferase, and CMV-Renilla luciferase were treated with tunicamycin (‘TUN’, 0.1 µg/mL) for 16 h. At this time, the cells were pulse-labeled with with ^35^S-amino acids for 30 min and chased for 2.5 h. The media were collected and cells were lysed and analyzed by immunoprecipitation with anti-insulin (the proinsulin band is shown). **D.** The ratio of simultaneous BiP-firefly to CMV-renilla luciferase activities are quantified from the experiment shown in panel C. **E.** At 2.5 h of chase from two independent experiments like that shown in panel C, the percent of total proinsulin recovered in the medium is expressed as mean ± s.d.

Proinsulin misfolding, as occurs in *MIDY*, is associated with ER stress [5], [15], [16], [17], [35]. We asked whether ER stress and ER stress response can by itself efficiently block wild-type proinsulin trafficking. We found that co-expression of the Hong Kong-null mutant of α_1_-antitrypsin, which itself is blocked in the secretory pathway (Fig. 7A *upper panels*) and is known to induce ER stress [49], could not block concomitant secretion of wild-type proinsulin (Fig. 7A lower panels). ER stress response pathways (PERK□phospho-eIF2α□ATF4; ATF6 activation; and Ire1□XBP1 mRNA splicing) positively regulate BiP transcription [50], [51], [52], [53]; and indeed, overnight treatment of cells with 0.1 µg/mL tunicamycin activates ER stress response as confirmed by a BiP promoter-firefly luciferase reporter [54] (Fig. 7D). Nevertheless, we found that this too could not block secretion of wild-type proinsulin (Fig. 7C, E).

**Figure 8:**
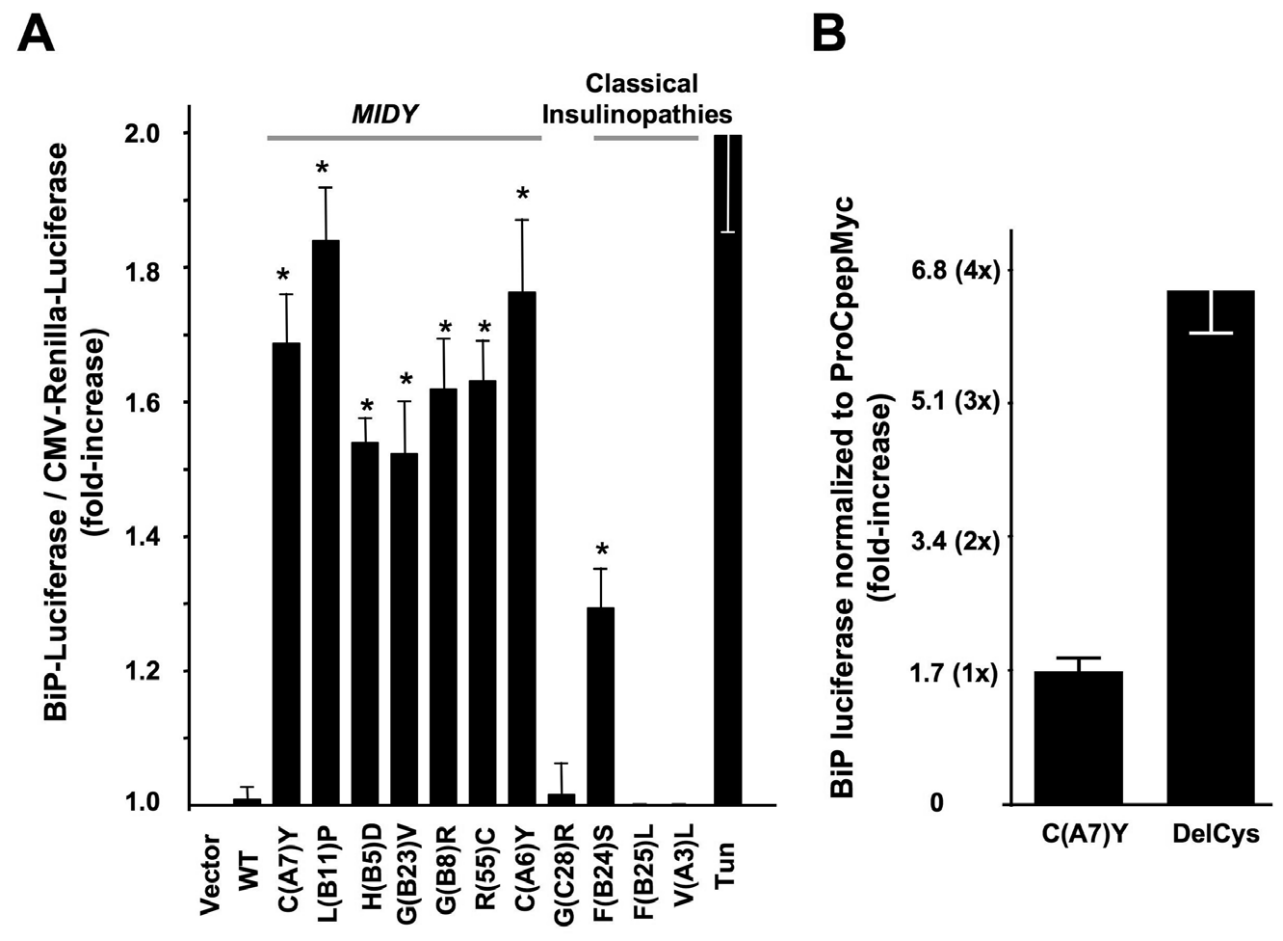
*MIDY* mutants induce ER stress in pancreatic beta cells. **A.** Min6 cells were transiently transfected with the BiP promoter-firefly luciferase plasmid, the pRL-CMV-driven Renilla luciferase reference plasmid, and empty vector or proinsulin expression plasmids at ratio 1 **:** 2 **:** 5 (this ratio helps ensure that BiP-luciferase serves as a reporter from cells synthesizing exogenously expressed proinsulins). At 48 h post-transfection, the cells were lysed and a ratio of firefly/renilla luciferase was measured. The relative activity of the BiP promoter in cells expressing mutant proinsulins was compared to that in cells expressing wild-type (“WT”) proinsulin, which served as a negative control. In a separate co-transfection Min 6 cells were treated overnight with tunicamycin (‘Tun’, 1 µg/mL), which served as a positive control for ER stress induction. Results are expressed as mean ± s.d. from at least four independent experiments. *<0.05 compared with proinsulin-WT **B.** Min6 cells were co-transfected to express BiP-luciferase and either hProCpepMyc-C(A7)Y or hProCpepMyc-DelCys. At 48 h after transfection, the cells were lysed and BiP-luciferase activity was measured along with the steady-state level of proinsulin mutants by Western blotting with anti-myc. Neither mutant is secreted by from the cells to the medium; note that DelCys is not deficient for induction of ER stress response.

Nevertheless, expression of *MIDY* mutants did induce ER stress in pancreatic beta cells. At 48 h after transfection of Min6 cells, each of the examined *MIDY* mutants induced BiP-luciferase activity relative to wild-type proinsulin (Fig. 8A). Neither expression of the proinsulin-G(C28)R variant nor the mutants classically associated with adult-onset diabetes [F(B25)L or V(A3)L] activated the ER stress response in pancreatic beta cells, whereas proinsulin-F(B24)S generated an ER stress response that was intermediate between that of wild-type proinsulin and that observed for *MIDY* mutants (Fig. 8A).

For a more precise analysis, we compared the ER stress response generated by proinsulin-DelCys to that of proinsulin-C(A7)Y as a positive control [5], [35], with BiP promoter-luciferase activity normalized directly to the steady-state levels of hProCpepMyc-DelCys *versus* hProCpepMyc-C(A7)Y in Min6 cells. We found that hProCpepMyc-DelCys caused significant ER stress (Fig. 8B) although it was ineffective as a dominant-negative inhibitor of wild-type proinsulin transport (Fig. 5). Whereas perturbation of the proinsulin folding pathway is central to the blockade of co-expressed wild-type proinsulin in *MIDY*, the data indicate that ER stress and ER stress response alone is not sufficient to account for the observed dominant-negative effects.

## Discussion

In the present study we have endeavored to understand the molecular basis for the onset of insulin deficiency in the syndrome of ***M***utant ***I*** *NS*-gene-induced ***D***iabetes of ***Y***outh (*MIDY*) [10], [11]. Proinsulin mutants causing the *MIDY* syndrome are distinct both from insulinopathies previously described as associated with adult onset-associated diabetes [3] and from recessive alleles that result in neonatal diabetes through reduced insulin expression [18]. Unequivocally, *MIDY* mutations cause misfolding of mutant proinsulin, blocking its progression to insulin [4], [5], [15] (this report, Figure S1), and activating ER stress response pathways [5], [15], [16], [17], [35] (this report, Fig. 8).

A prevailing hypothesis is that ER stress-mediated death of pancreatic beta cells with a resultant loss of beta cell mass triggers diabetes onset in *MIDY* [23]. Indeed, in *Akita* mice, there is little dispute that ultimately, after diabetes progresses, there is a significant loss of beta cell mass [29]; presumably this is the case in human *MIDY*, just as is the case in type 2 diabetes [26], [55], [56], [57]. However, decreased insulin production and diabetes in *Akita* mice, which is linked to inadequate wild-type proinsulin delivery to secretory granules [36], may occur before there is any loss of pancreatic beta-cell mass [30]. We now find that misfolding of the wild-type gene product is induced by the presence of the mutant gene product. Additionally, regardless of whether such mutations result in the loss, or creation, of an extra unpaired cysteine within proinsulin, we find that Cys residues play a critical role in the dominant-negative blockade of insulin production from the wild-type allele. Furthermore, while each of the *MIDY* mutants does cause ER stress and ER stress response, we find that ER stress and ER stress response alone cannot efficiently block insulin production from wild-type proinsulin and thus do not appear sufficient to account for the initiation of insulin deficiency.

Whereas a previous study using a two-dimensional non-reducing/reducing gel system failed to obtain evidence that the *Akita* mutant proinsulin promoted formation of aberrant disulfide bonds [34], we believe that a simpler, single-dimensional system of analysis results in a more robust assay [33], [36]; demonstrating that *MIDY* mutants are predisposed to form aberrant disulfide-linked protein complexes (Fig. 1A) and that *MIDY* mutants exhibit selective perturbation of the intracellular trafficking of co-expressed wild-type proinsulin (Fig. 3, 4C). This dominant-negative action precedes impairment of cellular ATP production or cell viability as it occurs in living cells that are translating and secreting proteins. Thus, we conclude that the following events are among the earliest in the molecular pathogenesis of *MIDY*. Specifically, shortly after expressing any of the *MIDY* mutants, the general ER export pathway for secretory proteins remains functional (Fig. 4C) even as wild-type proinsulin begins to be recruited in abnormal disulfide-linked protein complexes (Fig. 5B, C), impairing its delivery to secretory granules (Fig. 3A) and thereby impairing wild-type insulin secretion (Fig. 3B). This series of defects appears sufficient to account for insulin deficiency that is already in evidence on postnatal day 1 in *Akita* mice (for review, see [11]).

Our evidence favoring increased recruitment of wild-type proinsulin into aberrant disulfide-linked proinsulin-containing protein complexes, which serves to entrap wild-type proinsulin within the ER, is highly reminiscent of results from recent studies perturbing endogenous level of ERO1-beta (the endocrine pancreas-specific disulfide oxidase) [13]. In these knockout mice, a diabetic phenotype is linked to abnormally-increased proinsulin recruitment into interchain disulfide-linked adducts within the ER of pancreatic beta cells, accompanied by deficient proinsulin delivery to secretory granules for production of mature insulin [13]. These findings point strongly to the idea that perturbation of the proinsulin folding pathway, with or without mutations in the proinsulin coding sequence, is sufficient to trigger diabetes onset.

While it may be easy to dismiss as trivial the structural basis for aberrant intermolecular thiol attack by *MIDY* mutants that add or remove a cysteine (Fig. 2), it is more challenging to obtain data providing structural evidence for why non-cysteinyl proinsulin mutants are also predisposed to form aberrant disulfide-linked protein complexes *in vivo* (Fig. 1). In one particular example studied in detail, we examined insulin-G(B23)V chemically-synthesized from an insulin scaffold with native disulfide bonds already in place. Remarkably, insulin-G(B23)V has a largely native structure (Fig. 6) with essentially normal thermodynamic stability — even as the isolated B-chain bearing G(B23)V was essentially completely blocked in assembly with the A-chain to yield insulin. Since chain alignment leading to interchain disulfide bond formation is the crux of the chain-assembly assay, the data strongly suggest that there is an inability to create interchain disulfide pairing, and this kinetic blockade in the folding of the insulin chains (rather than instability of the native state) leads to enhanced cysteine thiol exposure that can promote intermolecular attack. Thus far, structural analyses of non-cysteinyl *MIDY* mutants remain quite limited. Nevertheless, from the experiments performed to date, we posit that *MIDY* mutations act as kinetic blocks in the folding pathway to proinsulin disulfide bond formation, resulting in free thiol availability that is a key to the molecular pathogenesis of *MIDY*.

In support of this hypothesis, we find that proinsulin-DelCys — despite being the most misfolded of all proinsulin mutants and completely blocked within the secretory pathway — cannot induce recruitment of co-expressed wild-type proinsulin into aberrant protein complexes (Fig. 5), cannot efficiently impair intracellular transport of co-expressed wild-type proinsulin (Fig. 4C) and thus cannot block insulin production or secretion (Fig. 3). These findings emphasize that at least one Cys residues is required for efficient dominant-negative blockade of insulin production from the wild-type allele, leading to an unfavorable chain of molecular events that results in progressive ER stress and beta cell failure. Unmistakably, ER stress and ER stress response are important consequences of proinsulin retention in the ER (Fig. 8A). However, ER stress and ER stress response alone cannot efficiently block production of insulin from wild-type proinsulin (Fig. 7). Thus it is not clear that ER stress, ER stress response, and loss of beta cell mass can account for initial pancreatic insulin deficiency that leads to the onset of diabetes in *Akita* mice, a model of *MIDY*.

In *MIDY*, the severity of phenotypes may be linked to the degree of folding (and secretion) disturbance [16], [17]. Of the mutations classically associated with adult-onset diabetes [1], [3], curiously, proinsulin-F(B24)S exhibits a more perturbed distal B-chain structure (Figure S3), a twofold decrease in insulin yield from the chain-assembly assay, a partial defect for secretion (Figure S1A), partial engagement in disulfide-linked protein complexes in the ER (Fig. 1), a partial dominant-negative effect on insulin production (Fig. 3), partial recruitment of wild-type proinsulin into disulfide-linked complexes (Fig. 5B), and partial activation of ER stress response (Fig. 8A). Loss of F(B24) may de-stabilize the native-like cluster of hydrophobic side chains near C(B19) and C(A20), decreasing the efficiency of disulfide pairing [58]. These findings appear consistent with a spectrum in the molecular pathogenesis of early-onset and late-onset diabetes caused by autosomal dominant *INS* gene mutations, ranging all the way to proinsulin-G(C28)R [7] which ultimately generates perfect human insulin lacking any mutation, does not use the *MIDY* mechanism (this report) and instead operates either through novel mechanisms involving the mutant C-peptide [15] or is coincidental to the pathogenesis of diabetes.

In summary, we have defined the molecular pathogenesis of *MIDY* as a syndrome in which mutant proinsulins use unpaired cysteine residues to recruit nonmutant proinsulins into disulfide-linked complexes, blocking insulin production that leads to insulin-deficiency, beta cell ER stress, and diabetes. Uncovering the earliest events in the molecular mechanism of the disease may help in identifying therapies designed to rescue proinsulin folding in the ER of pancreatic beta-cells.

## Materials and Methods

### Materials

Guinea pig anti porcine insulin antibody, Rat insulin radioimmunoassay (#RI-13K), human insulin-specific radioimmunoassay (#HI-14K) and human proinsulin specific radioimmunoassay (#HPI-15K) were from Millipore; rabbit anti-Myc antibody was from Immunology Consultants Labs; Zysorbin was from Zymed; [^35^S] amino acid mixture was from from ICN; DTT, Protein A agarose, and ‘RIA-grade’ BSA were from Sigma; 4-acetamido-4′-maleimidylstilbene-2,2′-disulfonic acid (AMS), Met/Cys-deficient DMEM (Dulbecco's modified Eagle's medium) and all other tissue culture reagents were from Invitrogen. Constructs encoding wild-type and Hong Kong-null α_1_-antitrypsin-myc were the kind gifts of Dr. R. Sifers (Baylor College of Medicine, Houston TX).

### Proinsulin mutagenesis and construction of hProCpepMyc and mouse ProCpepMyc

The mouse *Ins2* cDNA was amplified by RT-PCR from total RNA of isolated mouse islets, ligated into the pGEM T-vector and sequenced. *Ins2* cDNA was then cloned into pCMSGFP and the resulting plasmid was used as a template to introduce mutations associated with human diabetes, using the QuikChange site-directed mutagenesis kit (Stratagene). For proinsulin-DelCys, the C(A6) and C(A11) positions were mutated to methionine, C(A7) was mutated to tyrosine, and C(B7), C(B19) and C(A20) were each mutated to serine. Human preproinsulin cDNA was subcloned into pTarget (Promega) and the mutations were introduced as described above. A Myc-epitope was inserted into human or mouse C-peptide using following primers: human preproinsulin cDNA: 5′- GCAGGTGGAGCTGGGCGGGGGCCCTGAACAGAAGCTGATCTCAGAGGAGGACCTGGGTGCAGGCAGCCTGCAGCCCTTG-3′ and 5′-CAAGGGCTGCAGGCTGCCTGCACCCAGGTCCTCCTCTGAGATCAGCTTCTGTTCAGGGCCCCCGCCCAGCTCCACCTGC-3′; mouse preproinsulin cDNA: 5′-CAACTGGAGCTGGGTGGAGGCCCGGAACAGAAGCTGATCTCAGAGGAGGACCTGGGAGCAGGTGACCTTCAGACCTTG-3′ and CAAGGTCTGAAGGTCACCTGCTCCCAGGTCCTCCTCTGAGATCAGCTTCTGTTCCGGGCCTCCACCCAGCTCCAGTTG). Mutations were confirmed by DNA sequencing.

### Cell culture

INS832/13 rat insulinoma cells were kindly provided by Dr. C. Newgard (Durham, NC). Cells were cultured in RPMI 1640 supplemented with 10% FBS, 10 mM Hepes, 1 mM sodium pyruvate, penicillin-streptomycin and 50 µM 2-mercaptoethanol (Sigma). Min6 mouse insulinoma cells were cultured in DMEM supplemented with 10% FBS, penicillin-streptomysin and 50 µM 2-mercaptoethanol. 293T (human embryonic kidney-derived) cells were cultured in DMEM supplemented with 10% FBS and penicillin-streptomycin.

### Transfection of cells, metabolic labeling, and immunoprecipitation

293T cells were plated into 6 or 12-well plates 1 d before transfection. A total of 1–2 µg plasmid DNA was transfected using Lipofectamine (Invitrogen). Cells were pulse-labeled with ^35^S-labeled amino acids 48 h after transfection and chased for the times indicated. A proteinase inhibitor mixture was added to cell lysates and chase media. The samples were precleared with Zysorbin and immunoprecipitated as described in the text. Anti-insulin or anti-Myc immunoprecipitates were boiled for 5 min in gel sample buffer [1% SDS, 12% glycerol, and 0.0025% Serva Blue in 50 mM Tris (pH 6.8) with or without 100 mM DTT] and analyzed using tris-tricine-urea-SDS-PAGE under nonreducing or reducing conditions [59]. Immunoprecipitates of α_1_-antitrypsin were boiled for 5 min and resolved by conventional Laemmli SDS 10%-PAGE.

### Radioimmunoassay and Western blot analysis of secretion of mutant proinsulins

To examine secretion of mutant proinsulin beginning at 24 h after transfection, medium was replaced with serum free high-glucose DMEM plus 0.2% ‘RIA-grade’ BSA for 16 h. The media were collected and secreted proinsulins were measured using anti-rat insulin radioimmunoassay, which recognizes both human and mouse proinsulin and insulin. For hProCpepMyc constructs, transfected cell lysates and media samples were analyzed by Western blotting with anti-myc antibodies.

### Radioimmunoassay of secreted wild-type human proinsulin and insulin

293T cells were co-transfected with wild type human preproinsulin and mouse wild type or mutant preproinsulin at a DNA ratio of 1∶2. Beginning at 24 h after transfection, medium was replaced with high-glucose DMEM plus 10% FBS and further incubated for 16 h. The media were collected and secreted human wild type proinsulin was measured using human-specific proinsulin radioimmunoassay. Media collected from 293T cells transfected with wild type mouse proinsulin was used as negative control for radioimmunoassay specificity. INS-1 cells were similarly co-transfected using Lipofectamine 2000 (Invitrogen) at a DNA ratio of 1∶3. Cells were trypsinized 24 later and re-plated in triplicate wells. After 28 h incubation, media were then collected to measure human insulin specifically by radioimmunoassay.

### Alkylation of proinsulin Cys thiols

Immunoprecipitated proinsulin was resuspended and incubated in a buffer containing 2% SDS, 50 mM Tris pH 7.4 with or without 10 mM AMS for 1.5 h at 37°C. The reaction was stopped by boiling in SDS sample buffer plus 0.1 M DTT before analysis by Tris-tricine-urea-SDS-PAGE.

### Steady-State human insulin content of INS-832/13 cells transiently expressing mutant mouse proinsulin

INS-832/13 cells were transfected with mouse mutant proinsulin 1 day after plating using Lipofectamine (3 µg of plasmid DNA per well of a six-well plate). After 48 h, transfected cells were trypsinized, washed, resuspended in PBS and isolated by fluorescence-activated cell sorting. A total of 100 µl of acid-ethanol was used to extract insulin from each set of sorted INS-832/13 cells. Processed human insulin was measured in the extracts using a human insulin-specific radioimmunoassay.

### BiP promoter-driven luciferase assay

Min6 cells were plated into 24-well plates 1 d before transfection. Using Lipofectamine 2000 (Invitrogen), cells were co-transfected with pBiP-firefly-luciferase reporter plasmid [54] (provided by Dr. R. Kaufman,University of Michigan, Ann Arbor), CMV-renilla-luciferase plasmid (Promega), and human wild type or mutant proinsulin at a DNA ratio of 1∶2∶5, respectively. At 48 h post-transfection, cell extracts were prepared for the dual-luciferase reporter assay (Promega) with BiP-luciferase normalized to Renilla luciferase activity.

### Isolation and metabolic labeling of mouse pancreatic islets

Using *Akita* mice and wild-type littermates, islets were isolated and recovered overnight as described previously [36]. In each case, 50 islets were washed twice in prewarmed Met/Cys-deficient medium plus 1% BSA and 10 mM Hepes, pH 7.35. Islets were then pulse-labeled with ^35^S-labeled amino acids in the same medium for 20 min. After labeling, islets were directly immersed in lysis buffer containing a proteinase inhibitor mixture, immunoprecipitated with anti-insulin, and analyzed by Tris-tricine-urea-SDS-PAGE under both nonreducing and reducing conditions.

### Statistical analysis

Statistical analyses were carried out by ANOVA followed by Bonferroni's Multiple Comparison Test using GraphPad Prism 5. Data are presented as means±SD. A P value of <0.05 was taken as statistically significant.

### Synthesis of insulin analogs

Human insulin was obtained from Novo-Nordisk (Copenhagen, DK). H(B10)D-insulin and H(B10)D-*des*-octapeptide[B23-B30]-insulin were obtained from Eli Lilly Co. (Indianapolis, IN). Synthesis of variant B-chains, insulin chain assembly, and protein purification were performed as described [60], [61]. Selected B-chain analogs contained three “DKP” substitutions to prevent self-association of insulin [H(B10)D, P(B28)K, and K(B29)P]. V(A3)L-DKP-insulin was prepared by chain assembly. G(B23)V-DKP-insulin and F(B25)L-DKP-insulin were prepared by semisynthesis [62] beginning with H(B10)D-*des*-octapeptide[B23-B30]-insulin and corresponding C-terminal octapeptides (VFFYTKPT or GFLYTKPT, respectively). Preparation of F(B24)S-insulin has previously been described [48].

### ^1^H-NMR Spectroscopy

Spectra were obtained at 600 and 700 MHz in aqueous solution at pH 7–8 at 25° and 32°C and in 20% deuterioacetic acid at 25°C as described [63], [64]. DG/RMD calculations were performed as described [61].

## Supporting Information

Table S1 (1.28 MB DOC)

Figure S1 Test for secretion of mutant proinsulins known to be associated with human diabetes. 293T cells were transfected with vector alone or the same plasmid bearing wild-type preproinsulin (‘WT’) or preproinsulin missense mutants in which the described mutation is within the B-chain, the C-peptide, or A-chain. A. At 40 h post-transfection, cells were pulse-labeled with 35S-amino acids for 1 h, and then chased for 1 hour. The media (“M”) were collected and cells (“C”) were lysed. After immunoprecipitation with anti-insulin the samples were analyzed by nonreducing Tris-Tricine-urea-SDS-PAGE. B. Transfected 293T cells were divided in two equal portions. One portion was pulse-labeled with 35S-amino acids for 30 min (without chase) to examine new synthesis of proinsulins as measured by immunoprecipitation with anti-insulin followed by reducing Tris-tricine-urea-SDS-PAGE (lower fluorogram); the second portion was incubated with high glucose DMEM containing 0.2% BSA for 16 h and media were analyzed using a rat insulin radioimmunoassay that cross-reacts with proinsulins of all species (bar graph above). C. 293T cells transfected to express hProCpepMyc-WT (as described in Fig. 4 of the main text) or hPCpepMyc-C(A7)Y were incubated for 6 h in fresh medium before the media were collected and cells lysed. Equal fractions of cells and media were analyzed by SDS-PAGE, electrotransfer, and immunoblotting with anti-myc antibodies. The data highlight that the inability to recover MIDY mutant proinsulin in the media is unrelated to the specificity of insulin antibodies used for detection.
(6.75 MB TIF)

Figure S2 Aromatic spin systems of engineered monomer DKP-insulin and its analogs. Total correlation spectra (TOCSY) are shown of (A) parent DKP-insulin (for which the positions of highly reproducible NOEs are shown), (B) V(A3)L-DKP-insulin, (C) F(B25)L-DKP-insulin, and (D) G(B23)V-DKP-insulin. DKP-insulin contains two substitutions in the dimer interface [P(B28)K and K(B29)P] and one substitution in the trimer interface [H(B10)D]; its affinity for the insulin receptor is twice that of wild-type insulin. Spectra were acquired in each case at 25°C with TOCSY mixing time 55 ms. The chemical shifts of Y(A14) provides a sensitive marker of A-chain folding as it projects from the back surface of insulin, and the ortho-meta resonance of F(B24) provides a sensitive marker of folded state of the B-chain β-strand (B24–B28) due to its wild-type packing against C(B19) and L(B15), associated with an upfield resonance position in native-like structures (blue cross peaks in panels A–C). The insulinopathies V(A3)L and F(B25)L, which are classically associated with adult-onset diabetes, do not perturb the native fold of B- or A-chains; the MIDY substitution G(B23)V in a DKP-insulin in which disulfide bonds are already intact results in attenuation of resonances of F(B24) (red in panel D) and F(B25) (green in panel D) indicating local structural perturbation in the B-chain. Asterisks indicate altered cross-peak position of Y(B16) due to the absence of the F(B25) ring current (panel C) or altered positioning of the aromatic-rich β-strand (B24-B28) adjoining G(B23)V (panel D). Protein concentrations were 500–600 mM except for G(B23)V-DKP-insulin, which was diluted to 70 mM to avoid aggregation.
(10.45 MB TIF)

Figure S3 Solution structures of insulin analogs. (A) Ensemble of NMR-derived structures of DKP-insulin (Hua QX, et al., J. Mol. Biol. 264, 390–403 (1996)). The A- and B-chains are shown in light and dark gray, respectively. (B–D) Solution structures of V(A3)L-DKP-insulin (B), F(B24)S-insulin (C), and F(B25)L-DKP-insulin (D). In each case the mutant side chain is shown in red. Whereas V(A3)L is compatible with native-like structure in accord with results of X-ray crystallography (Wan Z-L, et al., Biochemistry 44, 5000-16 (2005)), F(B24)S destabilizes the C-terminal strand of the B-chain (Hua QX, et al., Proc. Natl. Acad. Sci. USA 90, 582-6 (1993)). (D) The solution structure of F(B25)L-DKP-insulin is essentially identical to that of DKP-insulin; differences in precision are likely to reflect extent of NMR analysis pursued under different conditions and not actual underlying differences in structure or dynamics. The solution structure of G(B23)V-DKP-insulin could not be obtained due to aggregation at protein concentrations amendable to the current NOESY analysis.
(10.47 MB TIF)
