# Correction: Mutant *INS*-Gene Induced Diabetes of Youth: Proinsulin Cysteine Residues Impose Dominant-Negative Inhibition on Wild-Type Proinsulin Transport

**DOI:** 10.1371/annotation/6d5e12f2-defc-48b5-84f6-43253f593a2a

**Published:** 2010-10-21

**Authors:** Ming Liu, Leena Haataja, Jordan Wright, Nalinda P. Wickramasinghe, Qing-Xin Hua, Nelson F. Phillips, Fabrizio Barbetti, Michael A. Weiss, Peter Arvan

The funding supporting Dr. Michael A. Weiss' contribution to this work was inadvertently omitted from the citation of funding. The funding acknowledgement should read: "This work was supported primarily by National Institutes of Health (NIH) R01 DK48280 (to P.A.), (NIH) R01 DK069764 (to M.A.W.), and also by a pilot and feasibility grant (to M.L.) and the Molecular Biology and DNA Sequencing Core of the Michigan Diabetes Research and Training Center (P60 DK20572). The authors gratefully acknowledge William and Dolores Brehm for their general support of diabetes research at the University of Michigan. The funders had no role in study design, data collection and analysis, decision to publish, or preparation of the manuscript."

