# Supplementary material for: Mutant INS-Gene Induced Diabetes of Youth: Proinsulin Cysteine Residues Impose Dominant-Negative Inhibition on Wild-Type Proinsulin Transport
Source: PLoS One. 2010 Oct 11;5(10):e13333. doi: 10.1371/journal.pone.0013333 (PMC2952628; doi:10.1371/journal.pone.0013333)
Supplement: Table S1 — (1.28 MB DOC) [file pone.0013333.s001.doc]

**Supplement Table 1.**

**Sequence Numbering and Syndromes linked to Dominant *INS* gene mutations**

**Signal Peptide (SP residues not shown in diagram below):**

1 11 21

MALWMRLLPL LALLALWGPD PAAA

| 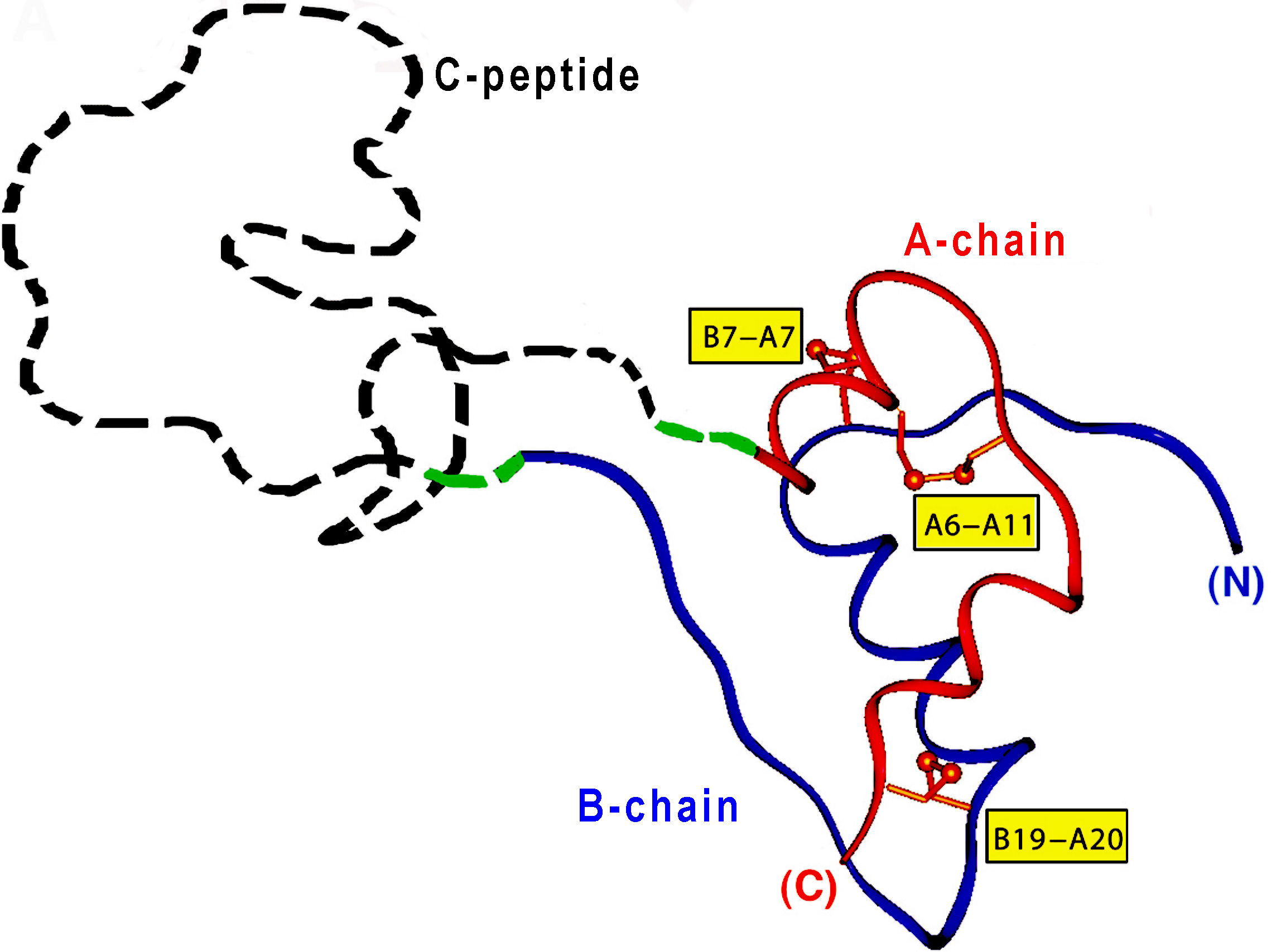 | *Monomeric proinsulin and insulin (PDB 4INS). Disulfide bonds shown in left panel; residues mutated in adult diabetes [V(A3), F(B24), and F(B25)] in right panel in red. Some of our structural studies employed substitution of H(B10), P(B28), and K(B29) (right panel in green)*.  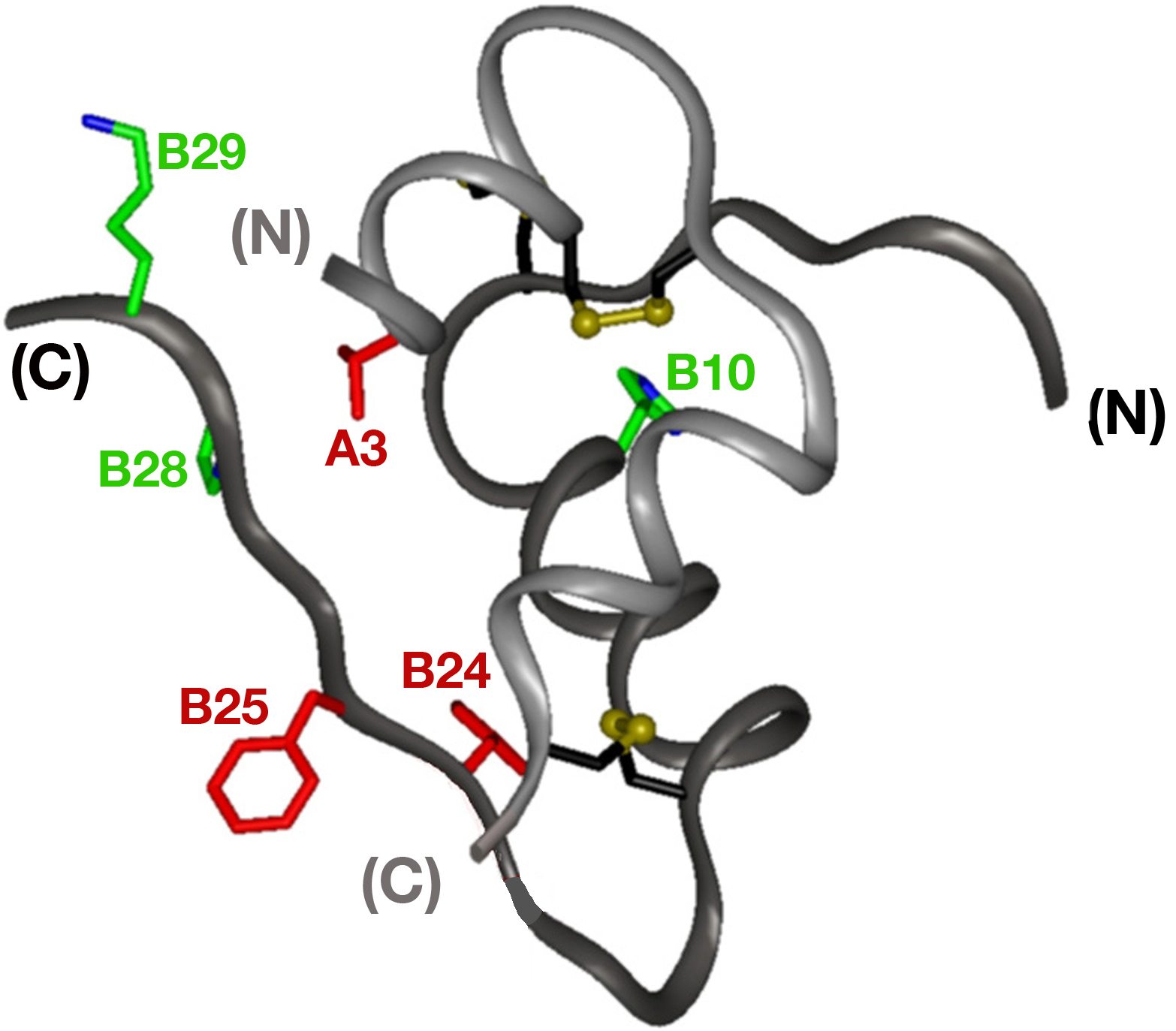 |
| --- | --- |

**B-chain (B) residues:**

1 11 21

FVNQHLCGSH LVEALYLVCG ERGFFYTPKT

**C-peptide/cleavage (C) residues:**

-2 1 11 21 31 +2

RR EAEDLQVGQV ELGGGPGAGS LQPLALEGSL Q KR

**A-chain (A) residues:**

1 11 21

GIVEQCCTSI CSLYQLENYC N

**Mutants Causing *MIDY*:**

Signal Peptide: R(S6)C or R(S6)H; A(S23)S; A(S24)D

B-chain: H(B5)D; L(B6)V or L(B6)P, G(B8)S or G(B8)R;

L(B11)P; LY(B15,16)H; C(B19)G; R(B22)Q; G(B23)V; F(B24)C

C-peptide/cleavage sites: R(C-2)C; G(C28)R; R(C33)C

A-chain: G(A1)C; C(A6)Y; C(A7)Y, C(A7)S, S(A12)C,

Y(A14)C, Y(A19)C, Y(A19)stop

**Mutants Associated with Adult-onset Diabetes:**

B-chain: F(B24)S, F(B25)L

A-chain: V(A3)L

**Mutants Associated with Hyperproinsulinemia:**

B-chain: H(B10)D

C-peptide/cleavage sites: R(C33)L, R(C33)P, R(C33)H
