# Supplementary figures and images for: Mutant INS-Gene Induced Diabetes of Youth: Proinsulin Cysteine Residues Impose Dominant-Negative Inhibition on Wild-Type Proinsulin Transport
Source: PLoS One. 2010 Oct 11;5(10):e13333. doi: 10.1371/journal.pone.0013333 (PMC2952628; doi:10.1371/journal.pone.0013333)

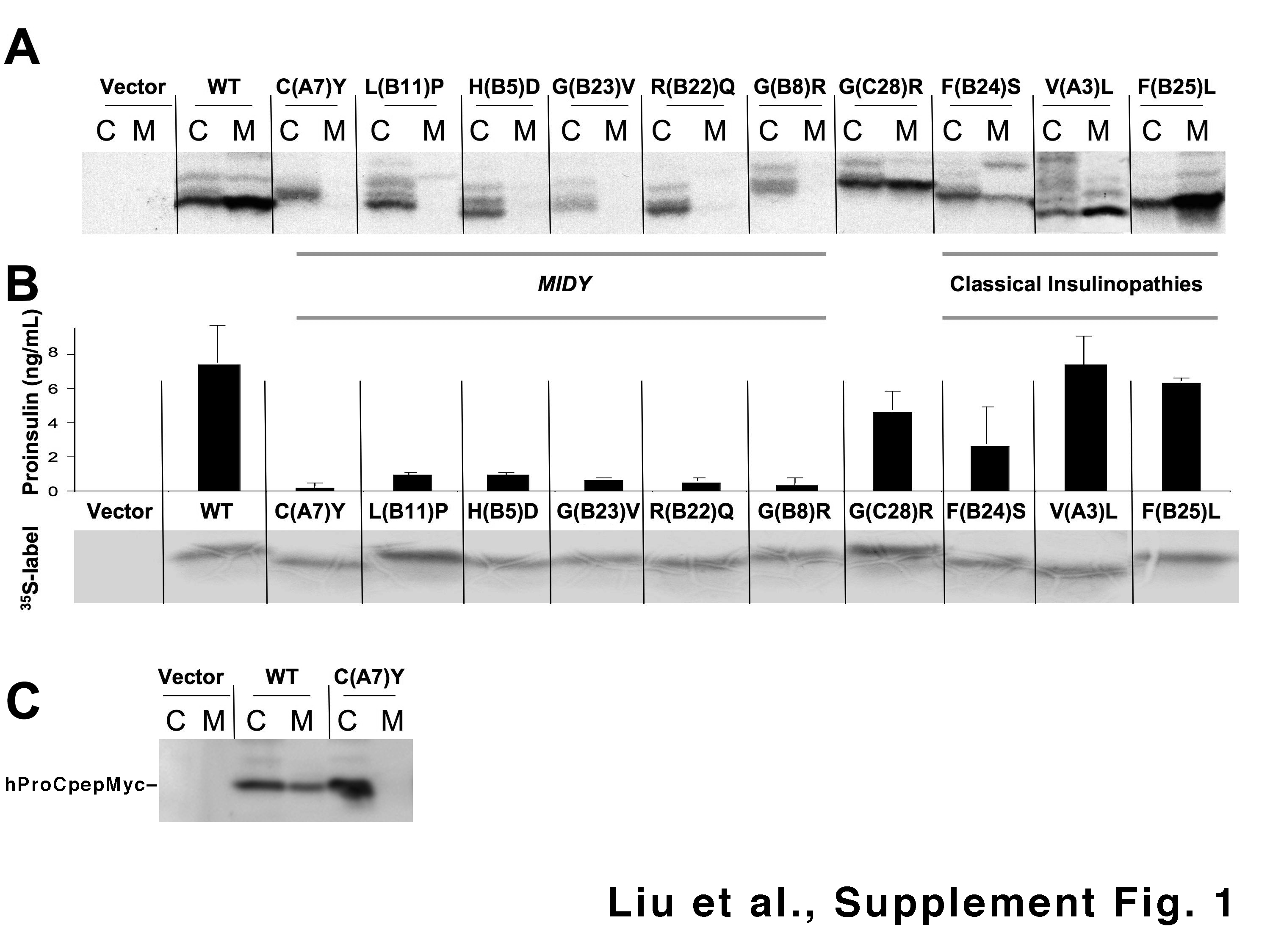

Supplement: Figure S1 — Test for secretion of mutant proinsulins known to be associated with human diabetes. 293T cells were transfected with vector alone or the same plasmid bearing wild-type preproinsulin (‘WT’) or preproinsulin missense mutants in which the described mutation is within the B-chain, the C-peptide, or A-chain. A. At 40 h post-transfection, cells were pulse-labeled with 35S-amino acids for 1 h, and then chased for 1 hour. The media (“M”) were collected and cells (“C”) were lysed. After immunoprecipitation with anti-insulin the samples were analyzed by nonreducing Tris-Tricine-urea-SDS-PAGE. B. Transfected 293T cells were divided in two equal portions. One portion was pulse-labeled with 35S-amino acids for 30 min (without chase) to examine new synthesis of proinsulins as measured by immunoprecipitation with anti-insulin followed by reducing Tris-tricine-urea-SDS-PAGE (lower fluorogram); the second portion was incubated with high glucose DMEM containing 0.2% BSA for 16 h and media were analyzed using a rat insulin radioimmunoassay that cross-reacts with proinsulins of all species (bar graph above). C. 293T cells transfected to express hProCpepMyc-WT (as described in Fig. 4 of the main text) or hPCpepMyc-C(A7)Y were incubated for 6 h in fresh medium before the media were collected and cells lysed. Equal fractions of cells and media were analyzed by SDS-PAGE, electrotransfer, and immunoblotting with anti-myc antibodies. The data highlight that the inability to recover MIDY mutant proinsulin in the media is unrelated to the specificity of insulin antibodies used for detection. (6.75 MB TIF) [file pone.0013333.s002.tif]

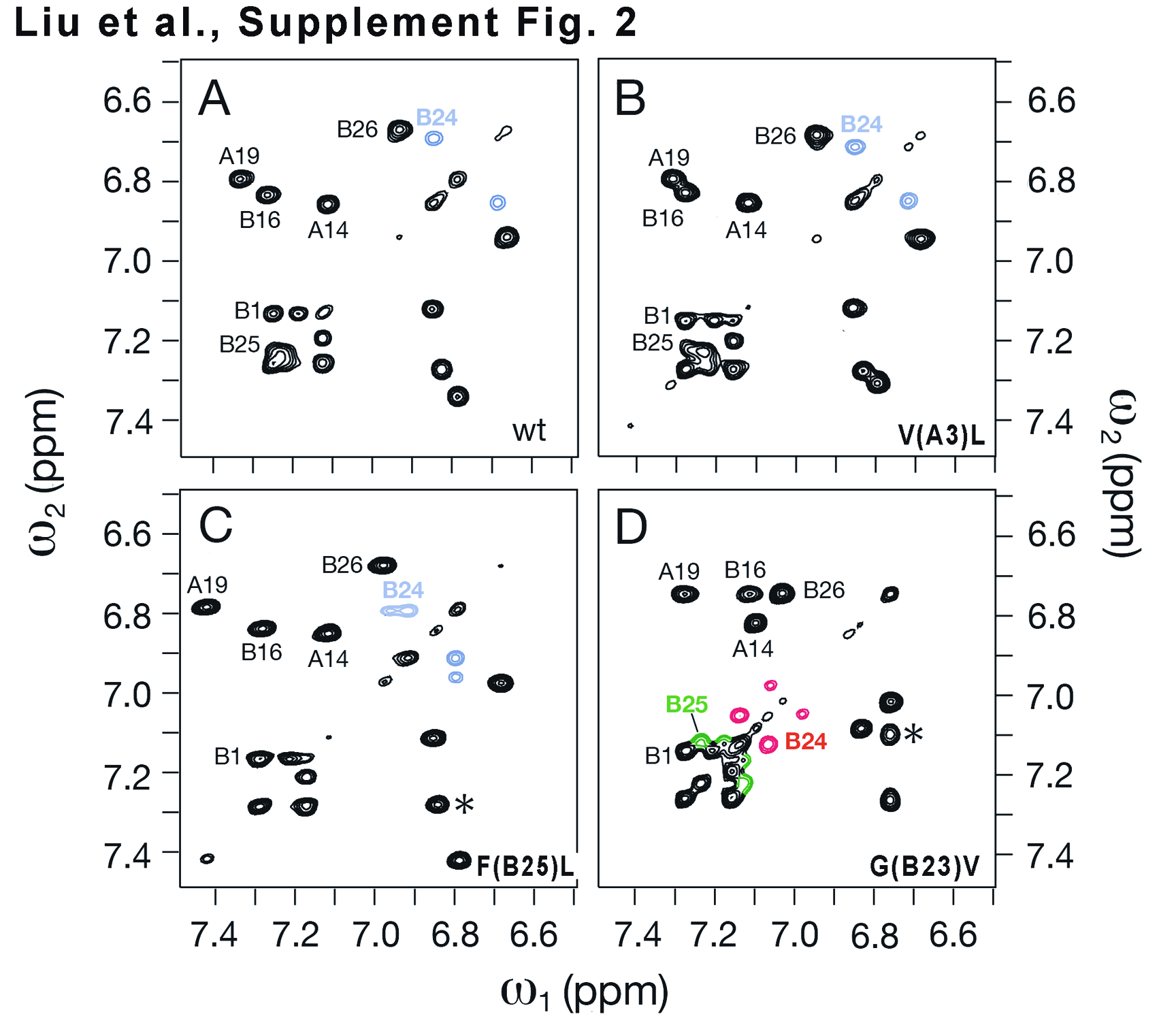

Supplement: Figure S2 — Aromatic spin systems of engineered monomer DKP-insulin and its analogs. Total correlation spectra (TOCSY) are shown of (A) parent DKP-insulin (for which the positions of highly reproducible NOEs are shown), (B) V(A3)L-DKP-insulin, (C) F(B25)L-DKP-insulin, and (D) G(B23)V-DKP-insulin. DKP-insulin contains two substitutions in the dimer interface [P(B28)K and K(B29)P] and one substitution in the trimer interface [H(B10)D]; its affinity for the insulin receptor is twice that of wild-type insulin. Spectra were acquired in each case at 25°C with TOCSY mixing time 55 ms. The chemical shifts of Y(A14) provides a sensitive marker of A-chain folding as it projects from the back surface of insulin, and the ortho-meta resonance of F(B24) provides a sensitive marker of folded state of the B-chain β-strand (B24–B28) due to its wild-type packing against C(B19) and L(B15), associated with an upfield resonance position in native-like structures (blue cross peaks in panels A–C). The insulinopathies V(A3)L and F(B25)L, which are classically associated with adult-onset diabetes, do not perturb the native fold of B- or A-chains; the MIDY substitution G(B23)V in a DKP-insulin in which disulfide bonds are already intact results in attenuation of resonances of F(B24) (red in panel D) and F(B25) (green in panel D) indicating local structural perturbation in the B-chain. Asterisks indicate altered cross-peak position of Y(B16) due to the absence of the F(B25) ring current (panel C) or altered positioning of the aromatic-rich β-strand (B24-B28) adjoining G(B23)V (panel D). Protein concentrations were 500–600 mM except for G(B23)V-DKP-insulin, which was diluted to 70 mM to avoid aggregation. (10.45 MB TIF) [file pone.0013333.s003.tif]

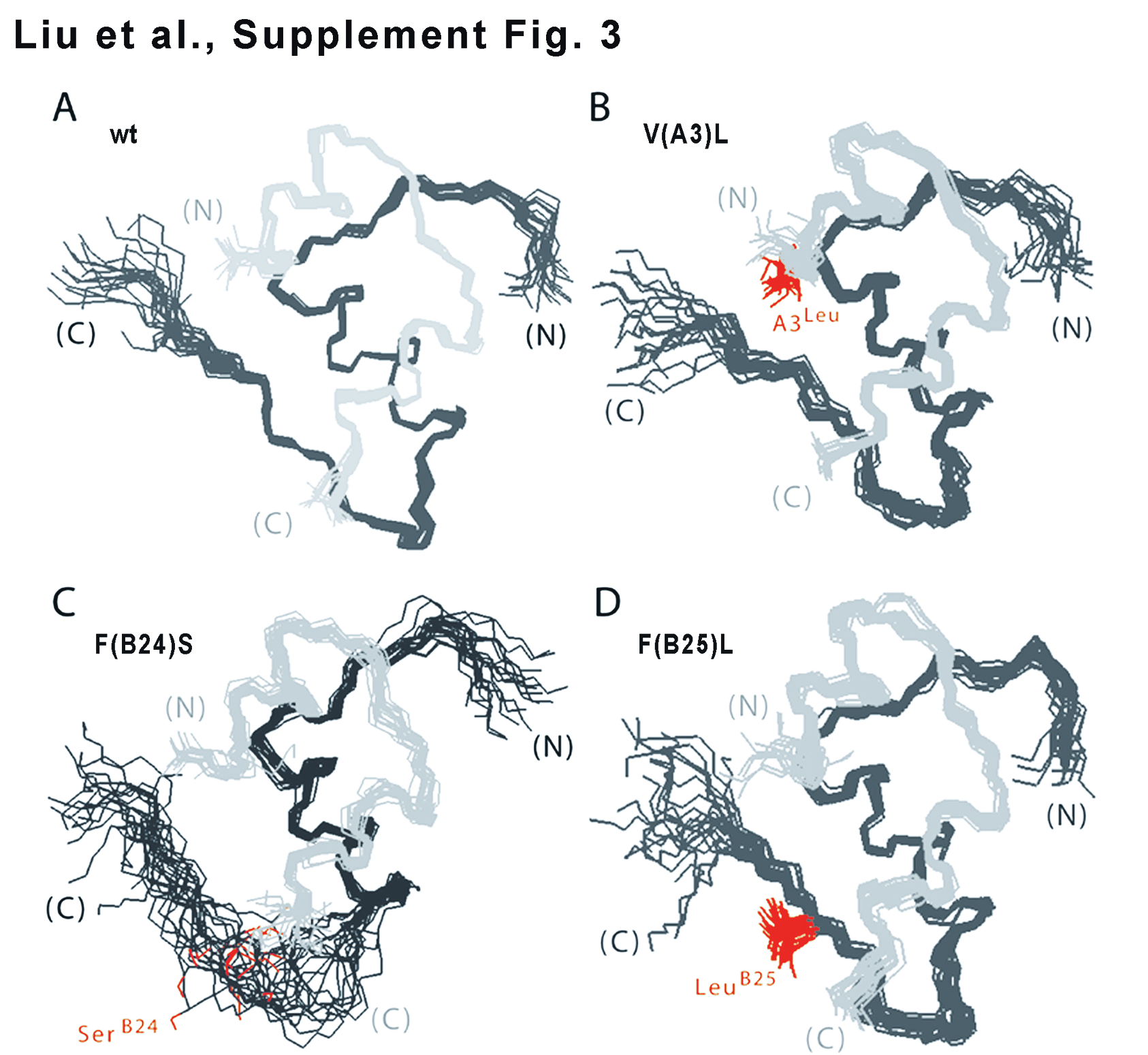

Supplement: Figure S3 — Solution structures of insulin analogs. (A) Ensemble of NMR-derived structures of DKP-insulin (Hua QX, et al., J. Mol. Biol. 264, 390–403 (1996)). The A- and B-chains are shown in light and dark gray, respectively. (B–D) Solution structures of V(A3)L-DKP-insulin (B), F(B24)S-insulin (C), and F(B25)L-DKP-insulin (D). In each case the mutant side chain is shown in red. Whereas V(A3)L is compatible with native-like structure in accord with results of X-ray crystallography (Wan Z-L, et al., Biochemistry 44, 5000-16 (2005)), F(B24)S destabilizes the C-terminal strand of the B-chain (Hua QX, et al., Proc. Natl. Acad. Sci. USA 90, 582-6 (1993)). (D) The solution structure of F(B25)L-DKP-insulin is essentially identical to that of DKP-insulin; differences in precision are likely to reflect extent of NMR analysis pursued under different conditions and not actual underlying differences in structure or dynamics. The solution structure of G(B23)V-DKP-insulin could not be obtained due to aggregation at protein concentrations amendable to the current NOESY analysis. (10.47 MB TIF) [file pone.0013333.s004.tif]
